# Structure Transformation and Morphologic Modulation of Supramolecular Frameworks for Nanoseparation and Enzyme Loading

**DOI:** 10.1002/advs.202207047

**Published:** 2023-04-14

**Authors:** Mingfeng Wei, Bao Li, Lixin Wu

**Affiliations:** ^1^ State Key Laboratory of Supramolecular Structure and Materials College of Chemistry Jilin University Changchun 130012 China

**Keywords:** 2D/3D interconversion, enzyme carrier, membrane separation, multiple interactions, supramolecular framework

## Abstract

Supramolecular framework (SF) encourages the emergence of porous structures with molecular flexibility while the dimension and morphology controls are less involved even though critical factors are vital for various utilizations. Targeting this purpose, two isolated components are designed and their stepped combinations via ionic interaction, metal coordination, and hydrogen bond into framework assembly with two morphologic states are realized. The zinc coordination to an ionic complex of polyoxometalate with three cationic terpyridine ligands constructs 2D hexagonal SF structure. A further growth along perpendicular direction driven by hydrogen bonding between grafted mannose groups leads to 3D SF assemblies, providing a modulation superiority in one framework for multiple utilizations. The large area of multilayered SF sheet affords a filtration membrane for strict separation of nanoparticles/proteins under gently reduced pressures while the granular SF assembly demonstrates an efficient carrier to load and fix horse radish peroxidase with maintained activity for enzymatic catalysis.

## Graphical abstract

Single‐layer 2D Supramolecular framework is constructed via combined coordination and ionic interactions and its stepped growth into 3D structure via hydrogen bond is realized reversibly. The multilayered 3D assembly in membrane shows precise size‐screened separation of nanoparticles and proteins while the granular state demonstrates the capability for enzyme fixation.

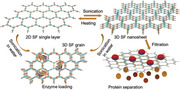

## 1 Introduction

Well‐dispersed monolayer assemblies with regular supramolecular framework (SF) structure, which are driven by intermolecular interactions, represent an emerging type of molecular porous architectures.^[^ ^1^ ^]^ Such kind of SF structure interlinks the properties of well‐known 2D nonporous structures like graphene and MXene and porous structures likes metal–organic frameworks (MOFs) and covalent–organic frameworks (COFs) at mono‐molecular level of thickness. As a result, the combined performance of SFs widens the porous characteristics and improves adaptability due to the utilization of increased dynamic driving forces. While bearing structural flexibility for processing into devices and materials conveniently, the regular pores within 2D assemblies in SFs also bring about important potentials in size control, adsorption, separation, drug carrier, and so forth.^[^ ^2^, ^3^, ^4^ ^]^ In contrast to mono‐/double‐ connecting bonds in the fabrication of various frameworks, the framework structures fabricating via more driving forces between molecular components become possible to bring more abundant features and functionalities.^[^ ^5^, ^6^ ^]^ Such a design also enables the driving forces in different dimensions to control the structure independently, and can provide a new assembly method from building units to bulk frameworks, which is different from the well‐known coordination/covalent frameworks.^[^ ^7^, ^8^ ^]^ In addition, the controlled structure growth from 2D layered framework to 3D framework and the reversible transformation can be realized through the dynamic intermolecular interactions. The reason is that accompanying the improvement of binding strength, either the sheet‐like shape of assemblies changes largely or the processing becomes difficult into membranes. The ionic interaction and its combination with host–guest interaction are once applied to fabricate 2D monolayer SF assemblies, which then grow into tight packing structure along with perpendicular direction.^[^ ^9^, ^10^ ^]^ Although the strategy exhibits collected advantages, the SF assemblies are still out of controlled, both on framework structures and the stepped utilization of multiple interactions.^[^ ^11^ ^]^ In general, a delicate modulation of the self‐assembly is decisive to achieve the specific architectures matching for the targeted applications.

Polyoxometalates (POMs) acting as a class of nanoscale polyanionic clusters have been used to construct supramolecular assemblies via the electrostatic interaction with cationic organic components. The diversity of organic building blocks and the dynamic tunability of electrostatic interaction enable the POMs to anchor in the formed assemblies and perform enhanced functions.^[^ ^12^, ^13^, ^14^ ^]^ The flexible ionic organic–inorganic frameworks bearing POM clusters as nodes driven by electrostatic interaction arouse the interest to build the POM‐mediated assemblies for collected potentials. Based on the rational composition of organic components and binding forces, regular porous assemblies of POMs are obtained to show unique properties in terms of separations via a simple filtration.^[^ ^15^, ^16^ ^]^ For example, the SF assemblies comprising of ionic POM complex are prepared for reversible oil/water separation and the size‐selective separation of sub‐nanoparticles with the small cut‐off value.^[^ ^17^, ^18^ ^]^ However, these known results are either existed as crystalline solid or limited to 2D SF structures, which are far from the wide needs in separations via a simple filtration process with enough stability and size screening range.^[^ ^19^, ^20^ ^]^ Therefore, solidified structure and extended frameworks favorable for the process become highly desired. Meanwhile, the successful structure extension will also enrich the library of SF assemblies. The metal coordination can dominate the binding direction of ligands delicately in comparing with the host–guest interaction while the electrostatic interaction plays a decisive role of controlling structural flexibility due to its nondirectional feature. The collection of the two interactions provides alike opportunity for the fabrication of more stabilized 2D SF assembly in principle although they have never been used for such a purpose.

**Scheme 1:**
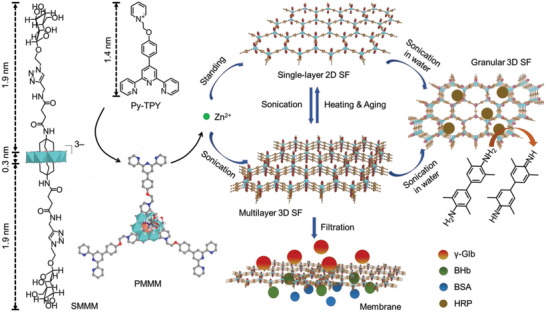
Schematic drawings of chemical structures of prepared mannose grafting cluster SMMM, cationic ligand Py‐TPY and their ionic complex PMMM, fabrication of single‐layer 2D SF assembly via Zn^2+^ coordination in DMSO/H_2_O (1:1), and transformation into multilayer 3D SF assembly via stepped growth under sonication and following interconversion between 2D and 3D structures in DMSO/H_2_O (1:1 in volume ratio), the 3D SF assembly membrane for nanoseparation of proteins, and the granular 3D SF for enzyme loading and catalysis.

As a result, only if the additional driving force is introduced on bridging nodes perpendicularly, the stepped growth of the 2D assembly into 3D porous structures in a controlled form will turn to be applicable. With this strategy, we herein report a gradual growth into layered 3D framework bearing inner layer and inter layer regular pores by using a polyanionic cluster as a multiply linking component, as shown in **Scheme** **1**. The obtained 2D SF structure bearing polyanionic cluster and cationic coordination complex is constructed via electrostatic interaction within 2D plane, which then grows up into 3D SF structures via hydrogen bond. The complicated assembly process can be well divided into separated procedures by modulating the assembly of small building units. For the known framework materials like MOFs, COFs, and hydrogen bond–organic frameworks (HOFs), etc., structural stiffness and granular morphology close to particles are the main features. Due to the electrostatic connection between building units, the present 2D and 3D framework assemblies can not only form very large ratio of area to thickness but also bear structural flexibility, which makes them easy form separation membrane with greatly reduced defects. In addition, by modulating the morphology of 3D SF into bulk state, the horseradish peroxidase (HRP) can be incorporated. Therefore, the membrane and carrier materials for separation of nanoparticles/proteins and assistance of the loading and catalysis of enzyme have been successfully achieved relying simply on the different 3D structures of the same supramolecular framework. These advantages not only provide a unique insight in the assembly process for the emerging area but also pave a steppingstone for functional applications.

## 2 Results and Discussion

### 2.1 Metal Coordination of Ionic POM Complex

**Figure 1:**
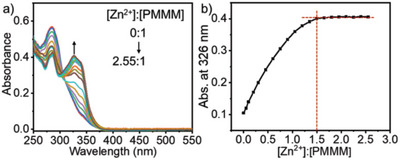
UV–vis titration spectra of Zn^2+^ and PMMM. a) UV–vis titration spectra of Zn^2+^ and PMMM in DMSO/H_2_O (1:1 in volume ratio) under the molar ratios ([Zn^2+^]:[PMMM]) of 0:1, 0.05:1, 0.1:1, 0.2:1, 0.3:1, 0.45:1, 0.6:1, 0.75:1, 0.9:1, 1.05:1, 1.2:1, 1.35:1, 1.5:1, 1.65:1, 1.8:1, 1.95:1, 2.1:1, 2.25:1, 2.4:1, 2.55:1, and b) corresponding absorption Job's plot at 326 nm with the molar ratio increase of [Zn^2+^]:[PMMM].

Based on the coordination interaction of terpyridine ligands with transition metal ions,^[^ ^21^, ^22^ ^]^ zinc ion is used to link the ionic complex PMMM for the formation of the SF assembly because of its linear binding with the ligand and nonionic interaction with the selected cluster. In the case of the coordination ratio of terpyridine to zinc ion at 2:1, the molar ratio of PMMM to zinc ion turns to be 2:3 for an ideal combination. The PMMM and ZnSO_4_ are separately dissolved in the mixed solvent of DMSO and H_2_O (1:1 in volume ratio) as a transparent solution with a concentration of 1.0 mg mL^−1^. By mixing them together at the given molar ratio for full coordination, faint yellow precipitate forms soon (Figure S10, Supporting Information) and is then filtrated and washed. Thermogravimetric analysis, organic and inorganic elemental analysis results give the consistent chemical composition corresponding to the stoichiometric binding ratio between PMMM and Zn^2+^ ion (Table S4 and Figure S11, Supporting Information). The coordination between zinc ion and terpyridine is characterized by X‐ray photoelectron spectroscopy (XPS). The decreased binding energy of Zn 2p^1/2^ and 2p^3/2^ by 1.2 eV reveals the occurrence of coordination (Figure S12a, Supporting Information).^[^ ^23^ ^]^ The vibration bands at 1607 and 1443 cm^−1^ ascribing to the stretching of C=N and C—N bonds in FTIR spectra move to 1600 and 1432 cm^−1^ after the addition of Zn^2+^, indicating the formation of Zn—N coordination bond which restricts stretching vibrations of bonds (Figure S12b, Supporting Information).^[^ ^24^, ^25^ ^]^ In comparison to the absorption of PMMM alone, the band at 326 nm in UV–vis spectrum sourcing from the metal‐to‐ligand charge transfer transition emerges, further proving the coordination (**Figure** **1**a).^[^ ^26^ ^]^ It should be mentioned that the addition of zinc ions into SMMM solution does not cause any precipitation, implying no specific ionic interaction between the two species bearing opposite charges.^[^ ^27^ ^]^ Under a fixed concentration of PMMM, a Job's plot of the coordination absorbance versus the molar ratio of [PMMM]/[Zn^2+^] from 1:0 to 2.55 is gained (Figure 1b). Accompanying by decrease of the absorption band at 284 nm, the absorption at 326 nm increases until the molar ratio reaches to 2:3, indicating the full coordination. Independently, a blue emission representing the coordination of zinc ion appears at 452 nm (Figure S13, Supporting Information).^[^ ^28^ ^]^ The Job's plot involving the luminescence also demonstrates the combination and the binding ratio of PMMM with Zn^2+^ at 2:3, suggesting the full complexation of ligand groups and the metal ion (Figure S14, Supporting Information).

### 2.2 Stepped Growth of SF Assembly from 2D to 3D

**Figure 2:**
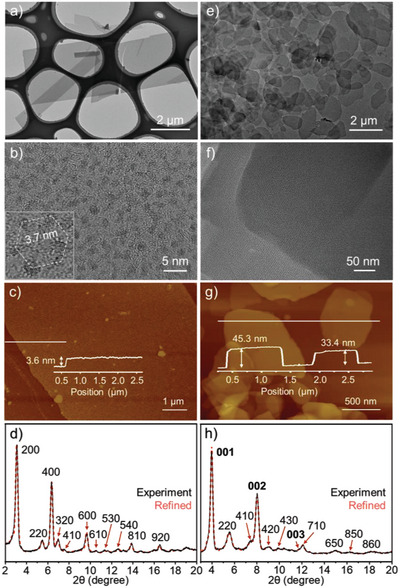
Characterization of 2D and 3D SFs. a) TEM image and b) its local magnification, c) AFM image and d) powder XRD pattern with Pawley‐refined dash curve of single‐layer 2D SF assembly prepared in DMSO/H_2_O (1:1 in volume ratio) on standing and simulations with *a* = *b* = 64.5 Å, Rwp = 10.56%, and Rp = 8.12%, while e,f) TEM images, g) AFM image, and h) powder XRD patterns with Pawley‐refined, dash curve of multilayer 3D SF assembly prepared in DMSO/H_2_O (1:1 in volume ratio) under sonication with *a* = *b* = 64.5 Å, *c* = 22.6 Å, Rwp = 6.99%, and Rp = 5.49%.

Based on the octahedral coordination mode of two terpyridine groups with one metal ion, the [2+3] type connection between PMMM and Zn^2+^ in their mixture solution will result in a hexagonal crosslinking. Transmission electron microscopy (TEM) images show that the formed supramolecular assembly possesses nanosheet‐like structure in size of several micrometers (**Figure** **2**a and Figure S15a,b, Supporting Information). The sharp edge and regular shape indicate the ordered packing within the assembly. The dark spots with heavy electron density in about 0.8–1.0 nm that is observed in the magnifying image can be attributed to the inorganic clusters (0.3 nm × 0.9 nm) (Figure 2b).^[^ ^29^ ^]^ Since the focused electron beam often cause position drifting of isolated clusters, the ordered pattern is not observed clearly. However, the distance between clusters at ≈3.7 nm and deviated hexagonal distribution can be recognized occasionally (Figure 2b inset). The distance is close to the sum of two Py‐TPYs in length with square crossing and diameter of a cluster. Atomic force microscopic (AFM) measurement provides the average height of the nanosheets about 3.6 nm (Figure 2c). The thickness matches well to the estimated molecular length of SMMM along the long axis, indicating the monolayer state of the assembly. The multiple diffractions in powder X‐ray diffraction (XRD) spectrum reveal the hexagonal framework structure within the single layer assembly (Figure 2d). A series of diffraction peaks at 2*θ* angles of 3.15°, 5.49°, 6.34°, 6.98°, 7.50°, 9.62°, 10.49°, 11.35°, 12.60°, 13.87°, and 16.51° fit to the diffraction indices of 200, 220, 400, 320, 410, 600, 610, 530, 540, 810, and 920, respectively. The assignment matches the lattice plane corresponding to the framework mode with side spacing ≈3.7 nm and the hexagonal aperture of ≈6.4 nm (Figure S15c,d, Supporting Information). It is noteworthy that the order in perpendicular direction is not observed and the reason can be explained that the packing of single layer assembly is not in good order, indicating well‐dispersed 2D framework assembly indirectly.

While the steric effect of bilaterally modified mannose groups on the cluster triggers the ordered packing of cationic ligand within 2D plane, the abundant hydrogen bonds also drive the connection along the perpendicular direction. Driven by sonication, the solution of 2D layered assemblies becomes more turbid and the single layer nanosheets become less and instead of that multilayered assemblies turn into the main assembly. Such an assembly structure can be obtained as well by a direct sonication to the solution of PMMM and Zn^2+^ in the direct preparation of 3D SF assembly. The TEM images show that the area of the nanosheets decreases but the thickness increases (Figure 2e,f and Figure S16a–c, Supporting Information). AFM images demonstrate the height increase of those nanosheets to 10–50 nm corresponding to several folds of a single layer (Figure 2g and Figure S16d–f, Supporting Information). Element mapping images focusing on the nanosheets demonstrate the presence of zinc, manganese, and molybdenum, indicating the existence of inorganic cluster and maintained coordination (Figure S17, Supporting Information). The diffractions in powder XRD demonstrate that the stepped assembly of isolated monolayer strengthens the structural order in the growth of lamellar structure (Figure 2h and Figure S18, Supporting Information). The series of diffraction peaks can be well assigned to the diffraction indices of 220, 410, 420, 430, 710, 650, 850, and 860 and all of them point to the hexagonal stacking within the layer.

Importantly, three additional diffractions at 2*θ* angles of 3.95°, 7.96°, and 11.86°, which do not appear in 2D assembly are observed and can be clearly attributed to the diffraction indices of 001, 002, and 003. The calculated *d*‐values 2.24, 1.11, and 0.75 nm with the ratio of 1:1/2:1/3 reveal the 3D packing structure deriving from 2D layered assemblies. As mannose groups locate outside the assigned framework plane, the hydrogen bonding between them is proposed to drive the ordered growth. According to the full length of mannose with the amide spacer in ≈1.9 nm and half thickness of the cluster in 0.15 nm, the head‐to‐head connection of the groups from adjacent layers gives far distance from the spacing from XRD pattern, which would cause unfavorable hydrogen bonding. Therefore, a full head‐to‐tail packing is inferred (Figure S19, Supporting Information), in which the mannose groups with amide chain adopt parallel packing with stronger interaction. The estimated overlapping length of ≈2.2 nm matches perfectly the layer spacing 2.24 nm from XRD data. The multiple hydrogen bonds help the structural stability in the perpendicular direction. The vibration bands at 3408, 3329, and 1666 cm^−1^ ascribing to stretching modes of hydroxyl —OH and amide —CONH— groups shift to 3398, 3289, and 1660 cm^−1^ in FTIR spectrum after the sonication treatment, indicating the formation of hydrogen bonds (Figure S20, Supporting Information).^[^ ^30^ ^]^ The bands attributing to triazole group also moves to low wavenumbers, suggesting its participating in the formation of hydrogen bonds. Based on the role of sonication in enhancing hydrogen bond to drive the formation of hydrogel structures,^[^ ^31^ ^]^ the same interaction between mannose groups with spacer under sonication is believed to trigger the transformation of 2D SF single‐layer into multilayer 3D SF assembly. To further verify the growth at the third direction deriving from hydrogen bond, a known hydrogen bond breaker urea is added into the solution of 3D SF assembly. The FTIR spectrum shows clearly band shifting to the 2D SF single‐layer state, indicating the decomposition of multilayer structure (Figure S21, Supporting Information).

### 2.3 Interconversions among Various SF Structures

The back process to the stepped formation of 3D SF assembly is achieved by raising temperature to 90 °C. The turbid solution becomes clear and then turbid again over time (Figure S22a, Supporting Information). It is seen that most of nanosheets decompose back into well dispersed single layers, and their size become larger again (Figure S22b, Supporting Information). The height diagram in AFM image confirms the single layer state of the 2D SF assembly in nanosheets (Figure S22c, Supporting Information). The XRD diffraction pattern shows that the diffraction peaks attributed to the ordered interlayer deposition disappear while the diffractions from hexagonal framework within 2D assembly maintain definitely (Figure S22d,e, Supporting Information). Following the first interconversion cycle, the repeated sonication to the assembly half an hour drives the single layer nanosheet to convert into multilayers for another time and the size becomes smaller. However, it is larger than the 3D SF nanosheets obtained by direct sonication, which can be ascribed to the initial larger monolayer assembly for the sonication induced accumulation along the perpendicular direction (Figure S23, Supporting Information). Due to the repeated diffractions attributing to the ordered interlayer stacking and the repeated parameters of the framework characterizations, it is obvious that the metal coordination structure still fit closely to the size and packing style from molecular components.^[^ ^32^ ^]^ Based on these structural within 2D SF plane maintains stable under the transformation conditions. By contrast, the hydrogen bond interaction between terminal mannose and spacer bearing amide and triazole groups regulates the 3D assembly and dissociation. (Figure S24, Supporting Information). Generally, the mannose group ended organic moiety on POM cluster does not maintain free for a longer time. Within a 12 h aging, the single‐layer 2D SF assembly further assembles into a multilayer structure automatically due to the presence of hydrogen bonding. Different from the sonication condition, however, the TEM characterization shows that the formed structure is less ordered and cannot be used for subsequent utilizations (Figure S25, Supporting Information).

**Figure 3:**
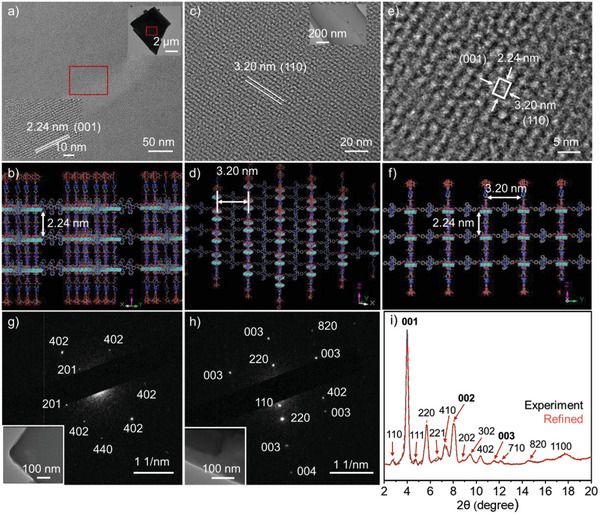
Characterization of granular 3D SF. a,c,e) TEM images and b,d,f) corresponding simulation diagrams of the crystal planes observed in TEM images at facets of 001 and 110 with and without *c*‐axis deviation, respectively, g,h) SAED patterns, and i) powder XRD patterns of 3D SF assembly prepared in water under sonication with Pawley‐refined dash curve with *a* = *b* = 64.5 Å, *c* = 22.4 Å, Rwp = 9.93%, and Rp = 6.82%.

In general, the intermolecular interactions strongly depend on the solvent types and the external conditions. Considering the overall hydrophilic/hydrophobic property of the building unit and formed ionic and coordination complex, when the ratio of DMSO increases, it weakens the hydrogen bond, as well as coordination and electrostatic interaction. As a result, the SF assembly will be no longer in good order. Though the SF assemblies do not dissolve in aqueous solution, the external conditions can improve the 3D packing structure. By sonicating in pure water, more ordered structure in the third direction (*c*‐axis) than that in the mixed solvent of DMSO and H_2_O is detected. The crystalline assemblies with size in several micro‐meters are observed in TEM images (Figure S26a, Supporting Information). The lattice fringes attributing to the different crystal planes clearly show in the local magnification. The spacing of the lattice fringe 2.2 nm ascribing to the indices of lattice plane (001) in XRD, is consistent with the proposed layer spacing of 3D structure based on molecular model (**Figure** **3**a,b), further proving the ordered growth of 2D SF layers along the third direction. In addition, the irregular‐shaped assembly formed from sonication allows the multiple observations to the lattice fringes at different angles. The layer spacing 3.2 nm agrees with the simulated value of interplanar spacing (110) in hexagonal stacking within the layer (Figure 3c,d). Magnification to the periods of both lattice spacings 3.2 and 2.2 nm at the same location is observed in accord with (110) plane in 3D SF structure (Figure 3e,f). In more detail, other lattice fringes between 2.2 and 3.2 nm are observed, which is attributed to the spacings of (110) at different angles. Typically, the clear spacings at 2.7 nm and 2.6 nm are well corresponding to the angles of 32° about 35° (Figure S26b–d, Supporting Information). In the selected area electron diffractions (SAED), diffraction spots consistent with XRD, including (003) and (004) indicating interlayer assembly, and (110), (220), (440), and (820) figuring out intrastratal hexagonal structure, are checked out. Meanwhile, the (201) and (402) diffraction patterns pointing to 3D structure appear distinctly, which further support the simulation to the proposed framework (Figure 3g,h). Compared with the mixed solvent, the assembly in water under sonication presents additional diffraction peaks that are assigned to (110), (111), (221), (202), (302), and (402), revealing the enhanced order of framework structure (Figure 3i and Figure S26e,f, Supporting Information). The pore size of 3D SF is also verified by nitrogen adsorption experiment (Figure S27, Supporting Information). A series of pore distributions can be recognized at about 2.2, 2.4, 2.7, 3.6, 4.8, and 6.3 nm. Based on the structural analysis of the present SF, the minimum aperture is consistent with the height of *c*‐axis in the layer, and the maximum aperture is consistent with the hexagonal aperture in the layer. Other measured channels correspond to pore sizes in different directions, which is consistent with the lattice fringes at different spacings observed in TEM images.

### 2.4 Multilayered 3D SF for Nanofiltrations

**Figure 4:**
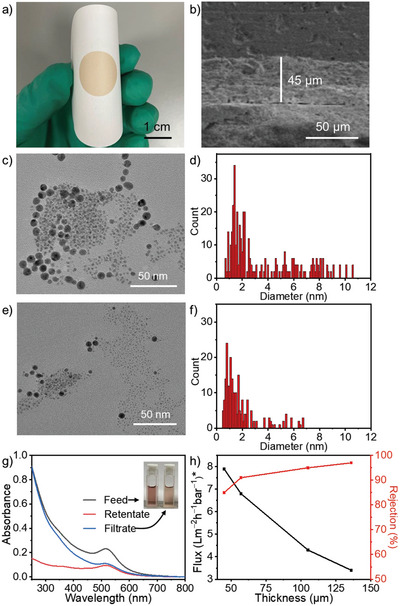
Separation of Au nanoparticles. a) Digital photograph and b) SEM cross‐section image of the prepared membrane, TEM images of Au NPs and corresponding statistic histograms of particle size c,d) before and e,f) after the filtration by membrane M136, and g) UV–vis spectra and the picture of the Au NPs before and after separation. h) Water flux and rejection efficiency plots of the particles versus the change of the membrane thickness. “*” means the flux value × 1000.

The SF assemblies bearing uniform pores and structural flexibility provide the possibility to form a membrane in nanofiltration. Compared with those known MOF/COF/HOF porous materials, the SF membranes possess uniform pores as well for filtration, which is favorable to use the regular channels. Meanwhile, the dynamic driving force helps the SF membrane for a certain degree of self‐healing for durability. The SF assemblies at single‐layer 2D nanosheet, multilayer 3D nanosheet, and nano‐granular like states are used for comparisons. The membranes are prepared by spreading these samples in DMSO/H_2_O (1:1 in volume ratio) on a commercial porous polymer‐supporting matrix under same reduced pressure (**Figure** **4**a). Since the separated nanoparticles are stable in water, DMSO is removed during the filtration process and is washed with water beforehand to ensure that the separation is carried out in water. The wet nanosheet assemblies with suitable flexibility deposit tightly and the unnecessary leakage can be blocked by increasing the quality of sample assemblies, thickness, and negative pressure (Figure 4b and Figure S28a, Supporting Information).^[^ ^17^ ^]^ For the membranes prepared at a pressure of −0.08 MPa, the components comprising the assemblies are no longer detected from the filtrate, indicating the stability of the SF structure against the separation conditions (Figure S28b, Supporting Information). Under the same experimental condition, the separation efficiency of the membranes for gold nanoparticles (Au NPs) in diameters about 9 nm is evaluated.^[^ ^33^ ^]^ It is found that the multilayer 3D nanosheet has the highest separation efficiency over 95%. The separation efficiency of single‐layer 2D nanosheet is about 90% while that of 3D nanogranular structure only comes to about 73% (Figure S29, Supporting Information). Considering the less strength and possible defects of single layer 2D SF assembly, and the unavoidable gaps between 3D SF mass upon processing, the multilayered 3D nanosheet assembly possess advantages both in structural strength and flexible spreading.

A mixture of trisodium citrate‐stabilized Au NPs with size distribution around 0.5–11.0 nm^[^ ^34^ ^]^ is used to evaluate the pore size and selectivity at a pressure of −0.08 MPa (Figure 4c,d). To build the relationship of the uniformity to the thickness of membrane, four membranes with the thicknesses of 45, 57, 105, and 136 µm (named as M45, M57, M105, and M136) are used (Figure S30, Supporting Information). After the separation of membrane M136, the Au NPs with size over 6.7 nm in the filtrate are eliminated completely since only nanoparticles in size smaller than that are observed (Figure 4e,f). This cut‐off value is a little bit larger than the diameter (6.4 nm) of inscribed circle on the hexagonal framework. Because the particles with size larger than the calculated pore size only occupies 4.6% in the filtrate, we infer that a few defects from flaws inter‐/intra‐SF assemblies are possible in the membrane. The decreased absorption at 517 nm in UV–vis spectrum of the sample solution after separation implies that large Au NPs are filtered out (Figure 4g). By calculating the proportion of different Au NPs sizes in statistical histogram from TEM images, it is found that the increase of thickness is favorable for a higher separation efficiency (Figures S31–S34, Supporting Information), but the flux is reduced proportionally as well. Within the range of membrane thickness from ≈45 to 136 µm, the flux decreases from 7902 to 3415 L m^−2^ h^−1^ bar^−1^ almost linearly, implying the high throughout and consistency of membrane quality (Figure 4h). Because Au NPs are hard nanosized objects that do not change shape during the filtration process, their cut off value suggests the fixed pore size in the membrane. To further demonstrate the recyclability of SF membrane in separations, the recovery rate of water flux (FRR) over multiple uses is tested. It is found that FRR values under different membrane thickness are all over 90% after five cycles (Figure S35, Supporting Information).

To demonstrate the extendibility of the 3D SF assembly in more separations, the framework membrane is used to screen proteins that are less purified via a filtration technique,^[^ ^35^ ^]^ by providing a convenient and low time saving post treatment.^[^ ^36^ ^]^ We conduct the separation of a series of proteins using the membrane M136. Bovine hemoglobin (BHb, 64.5 kDa) in size 6.4 nm × 5.5 nm × 5.0 nm,^[^ ^37^ ^]^ bovine serum albumin (BSA, 66.4 kDa) in size 7.0 nm × 3.8 nm × 3.8 nm,^[^ ^38^ ^]^ and *γ*‐globulin (*γ*‐Glb, 155–160 kDa) in size 15.6 nm,^[^ ^39^ ^]^ and selected as model proteins for the separation via filtration under mildly reduced pressure. The particle sizes of these three proteins in the separation solutions are initially examined by DLS (Figure S36, Supporting Information). The average diameters of BHb and BSA are about 5.0 nm, and the average particle size of *γ*‐Glb is about 10.0 nm. For the sample solutions of isolated BHb and BSA, both rejection efficiency values are lower than 2%, indicative of that most of the two proteins pass through the membrane. In contrast to this, the retention efficiency of *γ*‐Glb is over 95% in the first separation, demonstrating that the protein has been screened according to its 3D size (Figure S37, Supporting Information).

**Figure 5:**
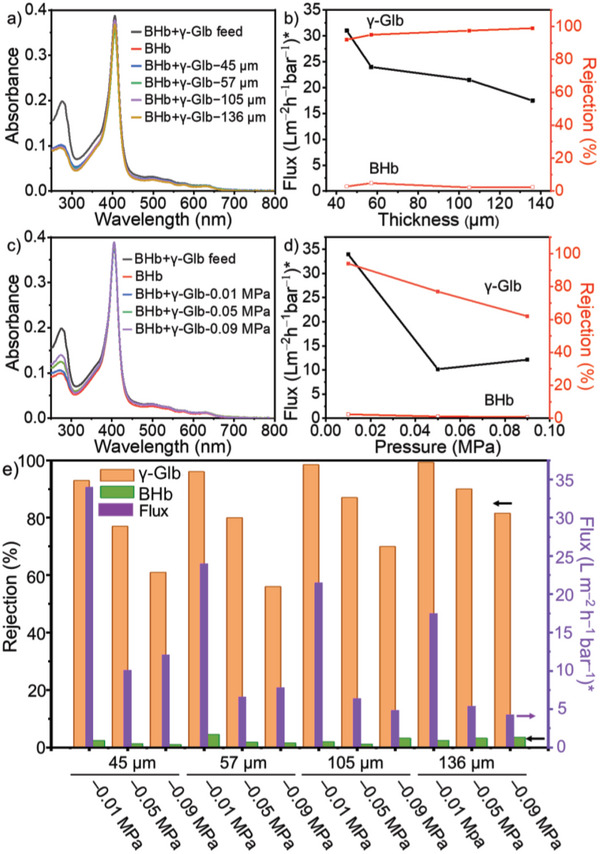
Separation of proteins. a) UV–vis spectra of BHb and *γ*‐Glb mixed solutions before and after separation by membranes with different thicknesses at a pressure of −0.01 MPa, b) plots of water flux and rejection efficiency of *γ*‐Glb versus the thickness of membrane at a pressure of −0.01 MPa, c) UV–vis spectra of BHb and *γ*‐Glb mixed solutions before and after separation by membrane M136 at different pressures, d) plots of water flux and rejection efficiency of *γ*‐Glb through M136 versus the negative pressure, and e) histogram of rejection efficiency and flux at different membrane thicknesses and pressures. “*” means the flux value × 100.

In the mixed solutions of BHb and BSA with *γ*‐Glb (C_BSA_:C_*γ*‐Glb_ = 2:1 and C_BHb_:C_*γ*‐Glb_ = 1:2), respectively, the DLS results show no apparent interaction induced aggregation behaviors (Figure S38a,b, Supporting Information). This means that the proteins in the mixture maintain their dispersed feature in the separation process. After the filtration to the protein mixtures via the 3D SF membrane, the particle sizes in the filtrates match in perfect to those belonging to BHb and BSA (Figure S38c,d, Supporting Information). During calculating the retention efficiency, to discern the influence caused by band overlapping between two proteins in UV‐vis spectrum, the work plots of absorbance versus the concentration of isolated proteins are employed (Figures S39–S41, Supporting Information). After eliminating the deviation, the rejection efficiency of BSA and BHb is still below 5%, and the interception efficiency of *γ*‐Glb is over 98% (Figure S42, Supporting Information). Apparently, the separation of the proteins is not affected by mixing them with others. Since the typical absorption of BHb at 405 nm has no overlap with other proteins, its mixed aqueous solution with *γ*‐Glb is used to evaluate the influence of the membrane thickness and the pressure on the separation of the proteins. At the fixed pressure of −0.01 MPa on bottom side of the membrane, the separation efficiency performs proportionally to the membrane thickness. When the membrane thickness is set at about 57 µm, the retention efficiency of *γ*‐Glb reaches about 95%. At the thickness of 136 µm, the retention efficiency reaches 99% while the retention efficiency of BHb maintains lower than 2%. In contrast, with the increase of membrane thickness from ≈45 to 136 µm, the flux decreases from 3400 to 1740 L m^−2^ h^−1^ bar^−1^ (**Figure** **5**a,b). In the case of fixed membrane thickness at 136 µm, accompanying by the increase of separation pressure from −0.01 to −0.09 MPa, the separation efficiency does not change parallelly. Instead, a gradually decreased retention efficiency of *γ*‐Glb from 99% to 82% occurs and the flux decreases greatly from 1740 to 460 L m^−2^ h^−1^ bar^−1^ (Figure 5c,d). The separation results versus the combined changes of membrane thickness and pressure are summarized systematically (Figure 5e and Figures S43–S49, Supporting Information). The thicker membrane is favorable for raising the retention of *γ*‐Glb but unfavorable for a good flux. The depressed pressure reduces both the retention efficiency and the flux of *γ*‐Glb. However, the penetration rate of BHb is less affected by membrane thickness and pressure. On the other hand, we find that with the increase of pressure, the protein flux decreases significantly. Such a phenomenon can be explained as case when the proteins are taken as the macromolecular assemblies with irregular shapes, which is similar to the deformation of polymers during filtration under pressure.^[^ ^40^ ^]^ Among the three proteins selected, BHb is nearly spherical while BSA is close to ellipsoid but *γ*‐Glb adopts an assembly in “Y” shape which consists of three subunits, two Fab and one Fc, respectively. The size of the *γ*‐Glb is about 15.6 nm, which is much larger than the pore size of the membrane. Although it can be retained, the size of its subunit Fab is about 4–5 nm,^[^ ^41^ ^]^ which is smaller than the pore size. Therefore, accompanying by the pressure increase, it is possible for the subunits of *γ*‐Glb which are connected with each other to enter different pores, resulting in clogging up of the pores and the decrease in flux. Meanwhile, it is observed that when the membrane is less than 60 µm, the flux at −0.09 MPa is slightly larger than that at −0.05 MPa, whereas when the membrane thickness is larger than 100 µm, the flux at −0.09 MPa is lower than that at −0.05 MPa. The reason can be explained as that the increase of pressure for a thinner membrane mainly causes more defects while for the thicker membrane, the increase of pressure has little effect on the defects but drive the structure deformation of proteins, leading to the blocking of pores by hanging on the framework bone. When the direction of the flow field is parallel to the direction of channels, a shear flow field emerges in front of the entrance accompanying by a parallel velocity gradient. Because the *γ*‐Glb subunits are smaller than the pore size, they can partially enter the pores driven by the elongational flow field.^[^ ^42^ ^]^

The hydrophilicity and static adsorption property of the membrane normally affect the separation process upon the fouling of proteins.^[^ ^43^ ^]^ The detected water contact angle of the SF membrane is close to 0°, indicating the membrane at the super‐hydrophilic state favorable to an enhanced antifouling property. This is reasonable since the covalently grafted mannose groups on top of 3D SF assembly can contribute to the hydrophilicity by adsorbing water molecules via hydrogen bond. In addition, the filling of water to the pores surrounding by frameworks can easily result in a superwetting surface through forming an ultrathin liquid layer on the membrane (Figure S50, Supporting Information).^[^ ^44^ ^]^ By taking the rejected BHb and BSA as the adsorbed leftover, the calculated adsorption values on the membrane are 3.0 and 18.0 µg cm^−2^, much lower than the adsorption mass of most polymer membranes (Figure S51, Supporting Information).

### 2.5 Granular 3D SF for Enzyme Loading and Catalysis

In contrast to the layered 3D SF suitable for filtration membrane, granular 3D SF bearing more solidified structure and morphologic merit performs a valuable carrier in adsorption behavior. The framework materials are known to stabilize the activity of enzymes which are loaded in the pores with the protected conformation. Therefore, the porous property that stabilizes the conformation becomes favorable for the fixation and functional applications of enzymes.^[^ ^45^, ^46^, ^47^ ^]^

**Figure 6:**
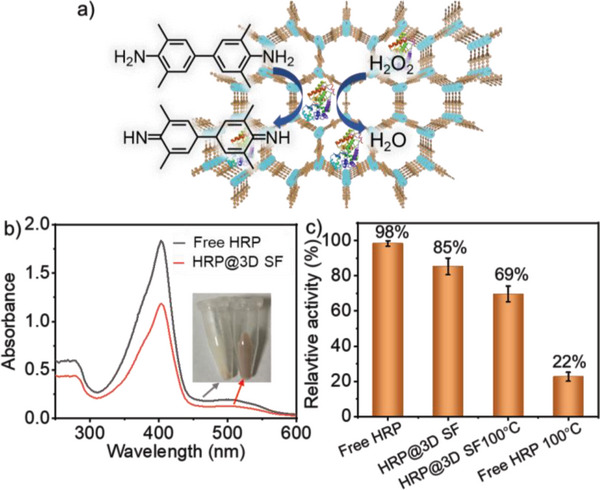
a) Schematic representation of the loaded HRP in 3D SF for the catalytic oxidation of TMB, b) UV–vis spectra of HRP before and after loaded by 3D SF, and c) relative activity of free HRP, HRP@3D SF, and after heating at 100 °C for 1.0 h.

However, the loading capacity is not high when only porous structures are loaded with enzymes, and additional binding conditions are needed. In contrast to general enzymes without specific interaction, a typical redox enzyme HRP bearing about 18% of mannose content is used as a model due to its hydrogen bond interaction with the 3D SF structure. The loading efficiency is characterized and the enzyme activity is evaluated for catalyzing the oxidation reaction of a regular substrate, 3,3′,5,5′‐tetramethylbenzidine (TMB) (**Figure** **6**a).^[^ ^48^, ^49^ ^]^ In general, the HRP possesses a size of ≈4.6 nm determined by DLS and a negative surface at about −9.6 mV measured by zeta potential in water (Figure S52, Supporting Information). Such features provide favorable conditions for HRP getting into the pores of SF via hydrogen bond between sugar groups from PMMM and HRP. To calculate loading efficiency, the corresponding work plot of absorbance versus the concentration of the isolated HRP is made (Figure S53, Supporting Information). It is found that 3D SF can load maximum of HRP up to 23% (wt) (Table S5, Supporting Information) while the enzyme structure seems maintained based on the unchanged UV–vis spectra before and after loading (Figure 6b). TEM images and XRD patterns (Figure S544a–c and Figure S55, Supporting Information) show no significant change with respect to the granular 3D SF before and after loading HRP, indicating the ignorant influence of the loading via hydrogen bond on the framework structure. On the other hand, iron element mapping images by means of energy‐dispersive X‐ray spectrum (Figures S54d and S56, Supporting Information) demonstrate that HRP has been loaded in the pores of 3D SF. To further determine the position of HRP in the framework, HRP@3D SF is characterized by laser confocal fluorescent microscope and small angle X‐ray scattering (SAXS).^[^ ^50^ ^]^ The HRP labeled with fluorescein isothiocyanate (FITC) is uploaded into 3D SF and the fluorescence deriving from dyed FITC‐HRP is found from the framework assembly, indicating the enzyme evenly dispersing in HRP@3D SF (Figure S57a, Supporting Information). On the other hand, the scattering peaks ascribing to 3D SF are observed in the SAXS experiment, and the result shows that no obvious change of the framework structure occurs after loading HRP. Because of the size of HRP smaller than the pores within framework, the loading does not affect the initial framework structure (Figure S57b, Supporting Information).

Following above understanding, the catalytic activity of the granular 3D SF after loading HRP is detected by the oxidation of substrate TMB. By taking the naked HRP as the reference, it is found that the enzyme activity decreases to about 85% after loading. The reason may be derived from that the mass transfer rate of substrate slows down the reaction somewhat in the framework in the case that local aggregation of enzyme is not considered. Therefore, the present 3D SF earns a comparable effect with respect to porous molecular materials like MOFs.^[^ ^51^ ^]^ We also evaluate the protection effect of 3D SF on the thermal stability of HRP. After treating free HRP and HRP@3D SF at 100 °C for 1.0 h, the HRP loaded in 3D SF still shows the activity about 81% in comparing to the sample without heating whereas the activity of free HRP has reduced to about 22% of original one (Figure 6c). This result indicates that HRP locate mostly in the pores of the framework structure. Due to the space limitation of the pore structure, the structure of the enzyme is stabilized and the activity of the enzyme is further maintained. As for the stability of the transformation of the framework structure, we also evaluate the stability of the 3D framework upon various pH, salt concentrations and temperatures in aqueous solution by means of UV‐vis spectra (Figure S58, Supporting Information). In order to verify the stability of the enzyme supported by the framework structure, the recycling catalysis is performed and the maintained conversions for oxidation of TMB confirm the structural stability of the enzyme (Figure S59, Supporting Information).

## 3 Conclusion

In conclusion, a single‐layer SF assembly with 2D hexagonal crosslinking structure is successfully constructed through the coordination bond and electrostatic interaction, by using an ion complex comprising of double mannose covalently grafting SMMM and cationic Py‐TPY in the presence of zinc ion. The initial layered framework structure is demonstrated to grow up stepwise in the third direction driven by the hydrogen bonding between the grafted mannose groups from adjacent layers in a form of head‐to‐tail under sonication in solution. Such a process builds a direct relation between 2D and 3D SF which is prepared discretely. Importantly, the 3D SF structure with a large area can be switched back to the 2D SF structure reversibly by means of a simple heating process. The precise design of composite building unit and the utilization of triple driving forces to control structural flexibility, connection rigidity, stepped association and dissociation make the SF assembly possess not only stabilized inner layer structure but also applicable in the formation of separation membrane for both hard nanoparticles and soft proteins via a simple suction filtration. The hydrophilic groups on top surface of the SF assembly and inner pores locating in the third direction allow the membrane to be superhydrophilic, which brings about the low fouling feature for general proteins during the filtration. At the same time, the uniform pore size ≈6.4 nm contributes a proportionate cut off value for the separation of larger proteins with a matched flux, which has not been involved up to date as far as we know. In addition, the unique inner surface of mannose groups within granular 3D SF assembly provides the opportunity for anchoring the enzyme bearing sugar groups via additional hydrogen bond. Thus, the 3D SF structure is proved to be the carrier of enzymes. As a result, the SF assembly becomes also the candidate to improve the thermal stability of enzyme with maintained catalytic activity. To be expected,^[^ ^52^ ^]^ more potentials of this type flexible porous assemblies can be developed in the near future.

## 4 Experimental Section

### Synthesis of Ionic POM Complex

In targeting the designed SF, an initial Anderson‐Evans type polyanionic cluster, [(C_4_H_9_)_4_N]_3_[MnMo_6_O_18_(C_4_H_8_N)_2_] (TBA_3_MM), was prepared following the published procedures.^[^ ^53^, ^54^ ^]^ Its subsequent amidation with succinic anhydride and then amino‐propyne gives an intermediate for the click reaction with 2‐azidoethyl mannose, yielding an organically modified cluster in tetrabutylammonium (TBA) salt, TBA_3_[MnMo_6_O_18_(C_19_H_30_O_11_N_5_)_2_] (abbrev. as TMMM). The TBA cations were then replaced with sodium ion to get Na_3_[MnMo_6_O_18_(C_19_H_30_O_11_N_5_)_2_] (abbrev. as SMMM) for further substitution of cationic ligand in the next procedure. The stepped modifications to the initial cluster and the chemical compositions of the obtained intermediates and product were carefully characterized by means of organic elemental analysis, ^1^H NMR, and mass spectra. The organic precursor 4′‐[4‐(2‐bromoethyloxy)phenyl]‐2,2′:6′,2″‐terpyridine was synthesized following similar procedures in the literature.^[^ ^27^, ^55^ ^]^ It was then used to prepare the cationic ligand 1‐(2‐(4‐([2,2′:6′,2″‐terpyridin]‐4′‐yl) phenoxy) ethyl) pyridine bromide (abbrev. as Py‐TPY) by the reaction with pyridine. The product was characterized by ^1^H NMR, ^13^C NMR, and matrix‐assisted laser desorption ionization time‐of‐flight (MALDI‐TOF) mass spectroscopy. The ionic complex was prepared by mixing SMMM with Py‐TPY in aqueous solution according to a full charge ratio through a simple ion replacement. The formed precipitate was filtrated and washed with water three times under sonication.

### Characterization of Ionic POM Complex

The obtained final product in a chemical formula of (Py‐TPY)_3_[MnMo_6_O_18_(C_19_H_30_O_11_N_5_)_2_] (abbrev. as PMMM) was characterized by ^1^H NMR and ^13^C NMR spectra, inorganic and organic elemental analysis. Because the cluster part is in a disk‐like shape with two mannose groups grafting on both sides covalently, three Py‐TPY ligand counter cations attach SMMM around equatorial side evenly. Due to the increased hydrophobicity caused by charge neutralization of Py‐TPY, the formed ionic complex becomes less soluble in aqueous solution. Therefore, in the following structural characterizations, mixed solvent of DMSO and H_2_O (1:1 in volume ratio) was used to get satisfied solubility. All the data are summarized in the Supporting Information (Schemes S1–S3, Figures S1–S9, and Tables S1–S3, Supporting Information).

## Conflict of Interest

The authors declare no conflict of interest.

## Supporting information

Supporting Information

## Data Availability

The data that support the findings of this study are available from the corresponding author upon reasonable request.
