# Supplementary material for: Structure Transformation and Morphologic Modulation of Supramolecular Frameworks for Nanoseparation and Enzyme Loading
Source: Adv Sci (Weinh). 2023 Apr 14;10(16):2207047. doi: 10.1002/advs.202207047 (PMC10238212; doi:10.1002/advs.202207047)
Supplement: Supplementary file 1 — Supporting Information [file ADVS-10-2207047-s001.pdf]

ADVANCED SCIENCE
Open Access

Supporting Information

for *Adv. Sci.*, DOI 10.1002/advs.202207047

Structure Transformation and Morphologic Modulation of Supramolecular Frameworks for Nanoseparation and Enzyme Loading

Mingfeng Wei, Bao Li and Lixin Wu**

Supporting Information

Structure Transformation and Morphologic Modulation of Supramolecular Frameworks for Nano-Separation and Enzyme Loading

Mingfeng Wei, Bao Li,* and Lixin Wu*

State Key Laboratory of Supramolecular Structure and Materials, College of Chemistry, Jilin University, Changchun 130012, P. R. China

Table of Contents

S1. Materials 2

S2. Measurements 2

S3. Synthesis of ionic complex (PMMM) 3

S4. Structural characterizations of synthetic intermediates and complexes 7

S5. Characterizations for metal coordination complexation 12

S6. Characterizations for morphology and structures of framework assemblies 14

S7. Size-selective separation of gold nanoparticles 22

S8. Size-selective separation of proteins 26

S9. Granular 3D SF for enzyme loading and catalysis 32

S10. References 37

S1. Materials. The general chemicals, D-Mannose, 4-dimethylaminopyridine [DMAP], 2-bromoethanol, Boron trifluoride etherate [$BF_3 \cdot O(C_2H_5)_2$], sodium azide, sodium methanolate, 4-hydroxybenzaldehyde (PHBA), 1,2-dibromoethane, 2-acetylpyridine, acetic anhydride (Ac_2O), N-ethoxycarbonyl-2-ethoxy-1,2-dihydroquinoline (EEDQ), 3,3',5,5'-Tetramethylbenzidine (TMB), 2-propynylamine, fluorescein isothiocyanate (FITC), succinic anhydride, ammonia hydroxide (29%), amberlite (IR 120 H^+), $HAuCl_4$, KOH, K_2CO_3, Na_2SO_4, NaCl, $NaClO_4$, $ZnSO_4 \cdot 7H_2O$, NH_4Cl and common organic solvents were purchased from Sinopharm Chemical Reagent Co., Ltd. Bovine serum albumin (BSA), Bovine hemoglobin (BHb), γ-globulin (γ-Glb) and Horseradish Peroxidase (HRP) were purchased from Beijing Solarbio Science & Technology Co., Ltd. All the commercially available products were used without further purification. Gold nanoparticles were synthesized referring to the method reported. [1,2] All the solvents were analytical grade and used as received. Doubly distilled water was used throughout the experiments. Silica gel (100−300 mesh) was employed in column chromatography.

S2. Measurements. ^1H NMR and ^{13}C NMR spectra were recorded on a Bruker Avance 500 MHz spectrometer by using tetramethyl silane (TMS) as internal reference (s= singlet, br= broad, d =doublet, t= triplet, q= quartet, m= multiplet). FT-IR spectra (KBr pellet) were collected on a Bruker Vertex 80 V spectrometer equipped with DTGS detector (32 scans) at a resolution of 4 cm^{-1}. Organic elemental analyses (C, H, N) were carried out on a Vario micro cube from Elementar. MALDI-TOF mass spectra were recorded on a matrix assisted laser desorption ionization (MALDI) time of flight (TOF) mass spectrometer (Bruker AutoflexTM speed TOF/TOF) equipped with a nitrogen laser (337 nm, 3 ns pulse). The matrix was trans-2-[3-(4-tert-Butylphenyl)-2-methyl-2-propenylidene] malononitrile (DCTB). The mass to charge ratio range during datum acquisition is from 700 to 2000 Da for reflection positive mode and 5K to 20K Da for linear positive mode. GC mass spectra were carried out by ion trap gas chromatography (GC) mass spectrometer (Thermo Fisher-ITQ1100) with electron impact (EI) ionization mode. Transmission electronic microscopic (TEM) images were obtained on a field emission electron microscope (JEOL JEM-2100F) with accelerating voltage of 200 KV without staining. Scanning electronic microscopic (SEM) measurement was performed on a JEOL JSM-6700F field emission scanning electron microscope. Atomic force microscopic (AFM) images were taken with a Dimension FastScanTM atomic force microscope from Bruker under ambient conditions. X-ray diffraction (XRD) data were recorded on a Rigaku SmartLab 3 X-ray diffractometer using Cu Kα1 radiation at wavelength of 1.542 Å. Static water contact angle (CA) measurements were performed with a Drop Shape Analysis System DSA10-MK2 from Krüss at ambient temperature. X-ray photoelectron spectra was carried out on an ESCALAB 250 spectrometer from Thermo with a monochromic X-ray source (Al Ka line, 1,486.6 eV) and the charging shift was corrected by the binding energy of C (1s) at 285.0eV. Laser confocal fluorescent microscope (FV1000) was used to determine the distribution of FITC-labelled HRP within 3D SF. SAXS measurements were performed on an Anton Paar instrument SAXSess mc2 with an X-ray wavelength of 1.542 Å and were carried out using a 1D detector at room temperature with an exposure time of 2 min.

S3. Synthesis of ionic complex (PMMM)

The synthesis of the ionic complex follows the route shown in Scheme S1−S3.

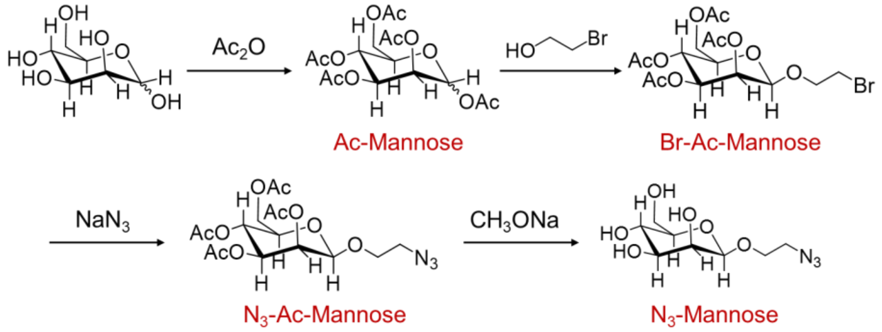

Scheme S1. Synthetic route of β-D-Mannopyranoside-2-azidoethyl (N_3-Mannose).

D-Mannopyranose-1,2,3,4,6-pentaacetate (Ac-Mannose). As shown in Scheme S1, DMAP (1.3 g, 11.1 mmol, 0.1 eq) was added slowly to a solution of D-Mannose (20 g, 111.1 mmol. 1 eq) and Ac_2O (78.6 mL, 8.3 mmol, 7.5 eq) in dry pyridine (110 mL). The reaction mixture was stirred overnight at room temperature under nitrogen and then diluted in the ethyl acetate (150 mL), washed with HCl (1M, 50 mL×5), NH_4Cl (50 mL) and brine (50 mL). The organic layer was dried over $MgSO_4$ and solvent was evaporated to dryness, giving the product (41 g, 105.1 mmol) as a sticky colorless oil in yield of 94.6%. ^1H NMR ($CDCl_3$, 500 MHz, 25°C): δ (ppm)= 6.06 (d, 1H), 5.33 (m, 2H), 5.23 (d, 1H), 4.24 (m, 1H), 4.09−4.05 (m, 2H), 2.15 (s, 3H), 2.11 (s, 3H), 2.07 (s, 3H), 2.13 (s, 3H), 1.98 (s, 3H).

β-D-Mannopyranoside-2-bromoethyl,2,3,4,6-tetraacetate (Br-Ac-Mannose). 2-bromoethanol (5.6 g, 45 mmol, 1.5 eq) was added to the solution of the Ac-Mannose (11.7 g, 30 mmol, 1 eq) in dichloromethane (80 mL). Boron trifluoride etherate (28.5 mL, 225 mmol, 7.5 eq) was added to the stirred solution at 0°C for 1 h and then at 35°C overnight. The solution was washed with water, aqueous sodium carbonate and saturated aqueous sodium chloride, respectively, dried over magnesium sulfate and evaporated in vacuo to give the crude product (12.02 g, 26.4 mmol) as a white powder in yield of 88%. ^1H NMR ($CDCl_3$, 500 MHz, 25°C): δ (ppm)= 5.35 (dd, 1H), 5.29 (t, 1H), 5.27 (dd, 1H), 4.88 (d, 1H), 4.27 dd, 1H), 4.13 (m, 2H), 3.93 (m, 2H), 3.52 (t, 2H), 2.16 (s, 3H), 2.11 (s, 3H), 2.05 (s, 3H), 2.00 (s, 3H).

β-D-Mannopyranoside-2-azidoethyl-2,3,4,6-tetraacetate (N_3-Ac-Mannose). Br-Ac-mannose of (11.0 g, 24.2 mmol, 1 eq) and sodium azide of (3.6 g, 55.0 mmol, 2.2 eq) which were previously dissolved in dimethyl formamide of 120 mL, were placed in a 200 mL round-bottomed flask and stirred overnight at 80°C under an oil bath. Ethyl acetate was added an appropriate amount to this reaction solution, the organic phase was washed sequentially with water, saturated aqueous sodium bicarbonate solution and saturated saline, dried with anhydrous sodium sulfate. Then the organic phase was filtered and the solvent was evaporated under reduced pressure and oil-like crude product was obtained. Recrystallization from ethanol gives 9.48 g of white solid product in yield of 97.6%. ^1H NMR ($CDCl_3$, 500 MHz, 25°C): δ (ppm)= 5.36 (dd, 1H), 5.30 (t, 1H), 5.29 (dd, 1H), 4.88 (d, 1H), 4.27 (dd, 1H), 4.13 (dd, 1H), 4.05 (ddd, 1H), 3.87 (m, 1H), 3.67 (m, 1H), 3.47 (m, 2H), 2.16 (s, 3H), 2.11 (s, 3H), 2.05 (s, 3H), 2.00 (s, 3H).

β-D-Mannopyranoside-2-azidoethyl (N_3-Mannose). The synthesis of N3-Mannose was followed by similar procedures reported in literature.[3] To a solution of N_3-Ac-mannose (3.2 g, 7.8 mmol, 1

eq) in dry MeOH (53 mL), freshly prepared NaOMe (1.0 M in dry MeOH, 1.2 mL) was added. The reaction was stirred at room temperature for 2 h. The reaction mixture was neutralized with an acidic resin (Amberlite IR 120 H^+) and the solvent was evaporated at reduced pressure, obtaining 1.85 g of product as a white solid in yield of 94.8%. ^{1}H NMR (D_2O, 500 MHz, 25°C): δ (ppm)= 4.93 (d, 1H), 4.00 (dd, 1H), 3.96−3.94 (m, 1H), 3.93−3.91 (m, 1H), 3.90 (dd, 1H, H3), 3.87−3.84 (m, 1H), 3.80-3.78 (m, 1H), 3.71−3.67 (m, 1H), 3.66−3.62 (m, 1H), 3.60 (dd, 1H), 3.59(s, 1H), 3.50−3.39 (m, 2H), as shown in Figure S1.

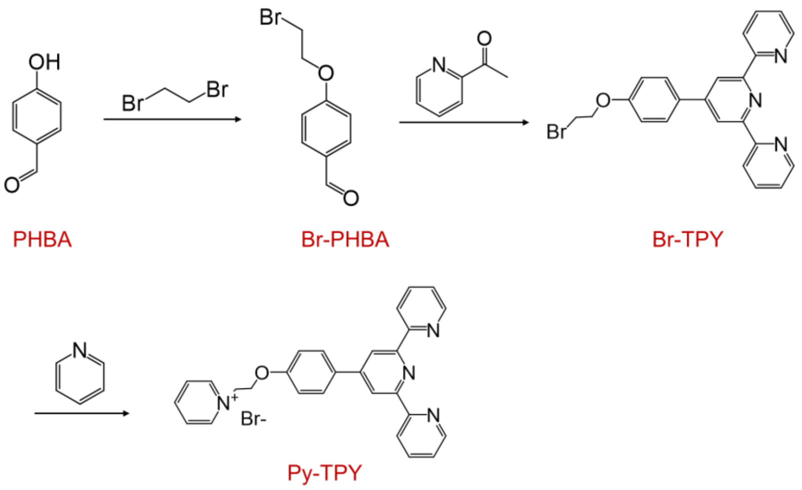

Scheme S2. Synthetic route of 1-[2-(4-((2,2':6',2''-terpyridin)-4'-yl)phenoxy)ethyl] pyridine bromide (Py-TPY).

4-(2-Bromoethoxy)benzaldehyde (Br-PHBA). As presented in Scheme S2, a mixture of PHBA (1.2 g, 10 mmol) and 1.2-dibromoetbane (3.7 g, 20 mmol) in dry acetone (20 mL) was heated under reflux for 5 h. Then, the reaction mixture was poured into water and extracted with dichloromethane. After drying over sodium sulfate, the solvent was removed under reduced pressure and the residue was purified on silica gel using a mixture (1:4) eluent of chloroform and petroleum ether to get 2.3 g of product in yield of 85.5%.^{1}H NMR (500 MHz, $CDCl_3$) δ (ppm)= 9.88 (s, 1H), 7.82-7.85 (d, 2H), 6.99-7.02 (d, 2H), 4.35-4.39 (m, 2H), 3.67-3.69 (t, 2H).

4'-[4-(2-Bromoethoxy)phenyl]-2,2':6',2''-terpyridine (Br-TPY). The synthesis of Br-TPY was followed by similar procedures reported in literature.[4] 2-acetylpyridine (1.0 mL, 9.2 mmol) was added to a solution of 4-(2-bromoethoxy) benzaldehyde (1.1 g, 4.6 mmol) in methanol (32 mL). To the solution, ammonia hydroxide (29%, 30 mL) and crushed KOH beads (0.37 g, 9.21 mmol) were added at the same time. A white precipitate appeared rapidly. The solution was then stirred at 50°C for 24 h. The resulting orange suspension was filtered. The off-white solid was rinsed with H_2O and CH_3OH and taken up in $CHCl_3$. After evaporating the solvent, the residue was purified by column chromatography on silica gel with a mixture eluent of CH_2Cl_2/MeOH/Et_3N (100/1/0.1) to give 935 mg of white solid (Br-TPY) in yield of 46.3%. ^{1}H NMR (500 MHz, $CDCl_3$) δ (ppm)= 8.79 (d, 2H), 8.76 (s, 2H), 8.72 (dt, 2H), 7.95 (d, 2H), 7.93 (td, 2H), 7.41 (ddd, 2H), 7.10 (d, 2H), 4.42 (t, 2H), 3.74 (t, 2H), as shown in Figure S2.

1-(2-(4-([2,2':6',2''-terpyridin]-4'-yl)phenoxy)ethyl) pyridine bromide (Py-TPY). Br-TPY (1.0 g, 2.3 mmol) was dissolved in pyridine solution (50 mL) and the solution was stirred at 90°C for 48 h. After cooled to room temperature, to the reaction was added ether (200 mL) to get a gray solid.

The product was obtained through the filtration under reduced pressure and washing with ether in yield of 95.8% (1.15 g). ^{1}H NMR (500 MHz, $CDCl_3$) δ (ppm)= 9.70 (d, 2H), 8.73 (d, 2H), 8.66 (d, 2H), 8.63 (s, 2H), 8.54 (t, 1H), 8.15 (t, 2H), 7.89 (t, 2H), 7.80 (d, 2H), 7.37 (ddd, 2H), 7.01 (d, 2H), 5.66 (t, 2H), 4.70 (t, 2H), as shown in Figure S3. ^{13}C NMR ($CDCl_3$, 500MHz, 298 K) (ppm)=159.18, 156.06, 155.44, 149.74, 149.22, 146.76, 145.97, 137.94, 131.06, 128.99, 128.47, 124.97, 121.48, 117.71, 115.91, 66.77, 60.44, as shown in Figure S3–4.

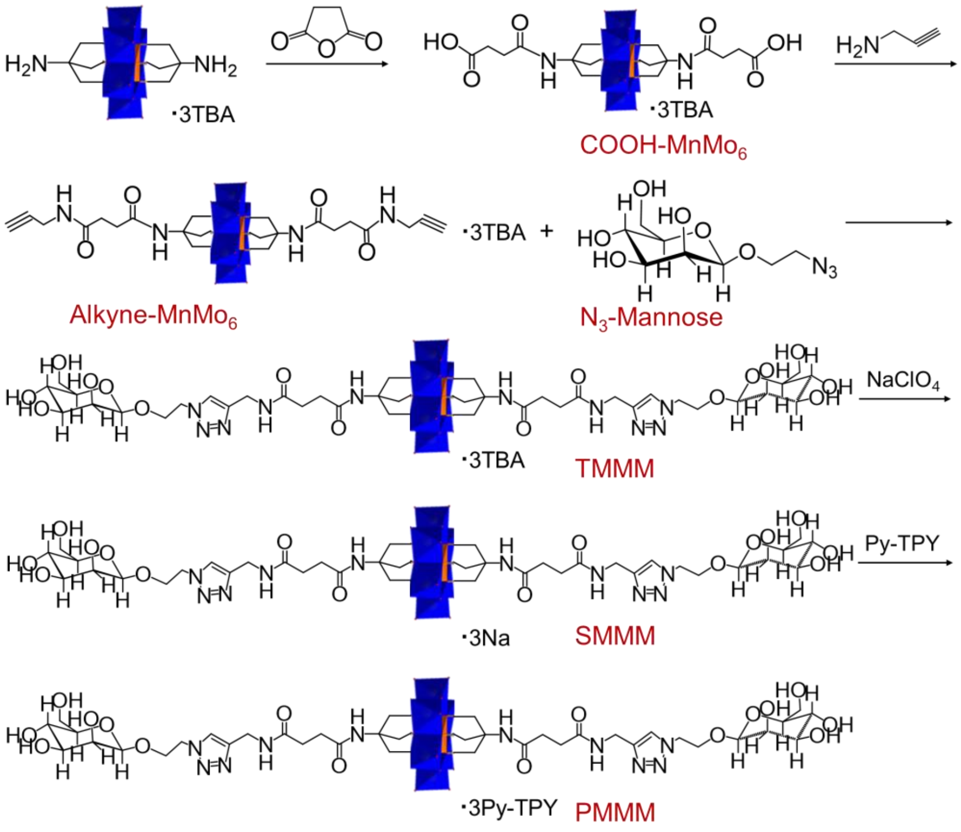

Scheme S3. Synthetic route of ionic complex molecule PMMM.

$[N(C_4H_9)_4]_3\{MnMo_6O_{18}[(OCH_2)_3CNHCO(CH_2)_2COOH]_2\}$ (COOH-MnMo$_6$). As shown in Scheme S3, the Anderson-type polyoxometalate, $[N(C_4H_9)_4]_3\{MnMo_6O_{18}[(OCH_2)_3CNH_2]_2\}$ (TBA_3MMM) (1.0 g, 0.55 mmol, 1 eq), which was synthesized according to the literature,[5] and succinic anhydride (1.2 g, 11.1 mmol, 20 eq) in CH_3CN (25 mL) was refluxed with stirring for 24 h. The orange solution was diffused with ether to produce orange crystals (1.3 g) within 24 h in yield of 49%. ^{1}H NMR (500 MHz, DMSO) δ (ppm)= 12.15 (s, 2H), 7.93 (s, 2H), 7.50 (s, 2H), 3.16 (t, 24H), 2.71(s, 4H), 2.31(s, 4H), 1.57 (m, 24H), 1.31 (m, 24H), 0.94 (m, 36H).

$[N(C_4H_9)_4]_3\{MnMo_6O_{18}[(OCH_2)_3CNHCO(CH_2)_2CONHCH_2CCH]_2\}$ (Alkyne-MnMo$_6$). The synthesis of Alkyne-MnMo$_6$ was followed by similar procedures reported in literature.[6] COOH-MnMo$_6$ (0.8 g, 0.4 mmol, 1 eq) and EEDQ (0.5 g, 1.9 mmol, 5 eq) were added into CH_3CN (25 mL) with stirring for 0.5 h at room temperate. Then, 2-propynylamine (77.5 µL, 1.1 mmol, 2.9 eq) was added and the solution was refluxed with stirring for 24 h. The orange solution was diffused with ether, producing 0.74 g of orange crystals within 24 h in yield of 88.4%. ^{1}H NMR (500 MHz, DMSO) δ (ppm)= 8.27 (s, 2H), 7.50 (s, 2H), 3.83 (s, 4H), 3.16 (t, 24H), 3.06 (s, 2H), 2.64(s, 4H), 2.27(s, 4H), 1.57 (m, 24H), 1.31 (m, 24H), 0.94 (m, 36H).

[N(C$_4$H$_9$)$_4$]$_3${MnMo$_6$O$_{18}$[(OCH$_2$)$_3$CNHCO(CH$_2$)$_2$CONHCH$_2$(C$_9$H$_{16}$O$_6$N$_3$)$_2$]$_2$} (TMMM). Alkyne-MnMo$_6$ (0.25 g, 0.1 mmol, 1 eq) was dissolved in DMF (10 mL) while N$_3$-Mannose (0.14 g, 0.56 mmol, 5 eq) was dissolved in H_2O (5 mL). Then the two aqueous solutions were mixed and $CuSO_4$ (64.0 mg, 0.26 mmol, 2eq) was added. The mixed solution was stirred for 0.5 h and the aqueous solution of NaAc (0.1 g, 0.5 mmol, 4 eq) was added with stirring for another 3 days. The green cloudy solution was added with ion-exchange resin (Amberlite IR 15 TBA$^+$), which was synthesized according to the literature. Filtration gave a clear orange solution. Most of water in the solution was removed under reduced pressure and the addition of ethyl acetate gave 0.28 g of orange solid product in yield of 93.1%. ^{1}H NMR (500 MHz, DMSO) δ (ppm)= 8.31 (s, 2H), 7.90 (s, 2H), 7.54 (s, 2H), 4.72 (s, 4H), 4.60−4.43 (m, 10H), 4.29 (s, 4H), 3.90 (s, 2H), 3.77 (s, 2H), 3.53 (d, 4H), 3.16 (t, 24H), 3.06 (s, 2H), 2.70(s, 4H), 2.31(s, 4H), 1.57 (m, 24H), 1.31 (m, 24H), 0.94 (m, 36H).

Na$_3${MnMo$_6$O$_{18}$[(OCH$_2$)$_3$CNHCO(CH$_2$)$_2$CONHCH$_2$(C$_9$H$_{16}$O$_6$N$_3$)]$_2$} (SMMM). TMMM (1.2 g, 0.46 mmol) dissolving in CH_3CN (5 mL) was added dropwise into a vigorously stirred CH_3CN solution (5 mL) with NaClO4 (5.7 g, 4.6 mmol). Then, the solution was stirred overnight to give an orange precipitate. Washing the precipitate twice with acetonitrile gave 0.89 g of the product SMMM in yield of 96.9%. 1H NMR (500 MHz, DMSO) δ (ppm)= 8.31 (s, 2H), 7.89 (s, 2H), 7.53 (s, 2H), 4.73 (s, 4H), 4.61−4.44 (m, 10H), 4.29 (s, 4H), 3.91 (s, 2H), 3.78 (s, 2H), 3.53 (d, 4H), 3.06 (s, 2H), 2.70(s, 4H), 2.31(s, 4H), as shown in Figure S5. ESI-MS (m/z): (H$^+$) [MnMo$_6$O$_{24}$] $(C_{38}H_{60}O_{16}N_{10})^{2-}$ (HMM^{2-}): 964.2, found: 963.8, [MnMo$_6$O$_{24}$] $(C_{38}H_{60}O_{16}N_{10})^{3-}$ (MM^{3-}): 642.5, found: 642.6. as shown in Figure S6. Elemental analysis calcd. for $(Na)_3MnMo_6O_{24}C_{38}H_{60}O_{16}N_{10}\cdot 4H_2O$ (NMM, 2068.5 g/mol): C, 22.06%; H, 3.31%; N, 6.77%, found: C, 21.92%; H, 3.580%; N, 6.54%.

(Py-TPY)$_3${MnMo$_6$O$_{18}$[(OCH$_2$)$_3$CNHCO(CH$_2$)$_2$CONHCH$_2$(C$_9$H$_{16}$O$_6$N$_3$)]} (PMMM). The obtained SMMM (0.8 g, 0.4 mmol) in mixture solvent (CH_3OH/H_2O in 1:1 v/v) (20 mL) was added dropwise into a vigorously stirred mixture solvent (CH_3OH/H_2O in 1:1, 10 mL) of Py-TPY (0.6 g, 1.2 mmol). The mixed solution was kept stirring overnight to give an orange precipitate which was washed twice with CH_3OH, giving 1.3 g of product PMMM in yield of 93.4% ^{1}H NMR (500 MHz, DMSO) δ (ppm)= 9.19 (d, 6H), 8.76 (d, 6H), 8.67 (d, 16H), 8.31 (s, 2H), 8.23 (t, 6H), 8.05 (t, 6H), 7.92 (d, 6H), 7.91 (s, 2H), 7.54 (t, 6H), 7.15 (d, 6H), 5.11 (t, 6H), 4.73 (s, 4H), 4.64 (t, 6H), 4.62−4.40 (m, 10H), 4.29 (s, 4H), 3.92 (s, 2H), 3.62 (s, 2H), 3.54 (d, 4H), 2.72(s, 4H), 2.32(s, 4H), as shown in Figure S7. ^{13}C NMR ($CDCl_3$, 500MHz, 298 K) (ppm)=173.44, 171.80, 159.05, 156.13, 155.45, 149.79, 149.31, 146.67, 146.03, 137.99, 130.95, 128.84, 128.57, 124.97, 123.85, 121.43, 117.83, 115.92, 100.31, 74.60, 71.33, 70.48, 67.33, 66.84, 65.43, 61.62, 60.70, 49.81, 34.88, 31.75, 29.65, as shown in Figure S8. Elemental analysis calcd. for $(C_{28}H_{23}ON_4)_3MnMo_6O_{24}C_{38}H_{60}O_{16}N_{10}\cdot 13H_2O$ (PTMM, 3456.229 g/mol): C, 42.40%; H, 4.53%; N, 8.89%, found: C, 42.39%; H, 4.52%; N, 8.91%.

S4. Structural characterizations of synthetic intermediates and complexes.

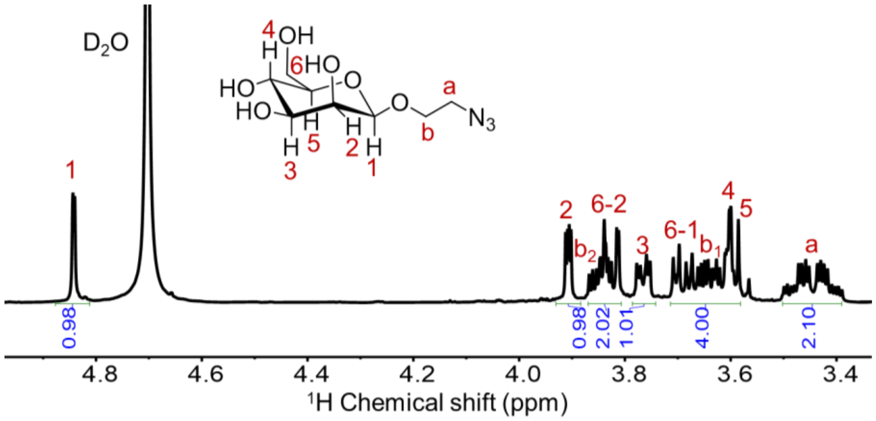

Figure S1. ^{1}H NMR spectrum (D_2O, 500 MHz, 298 K) of N_3-Mannose.

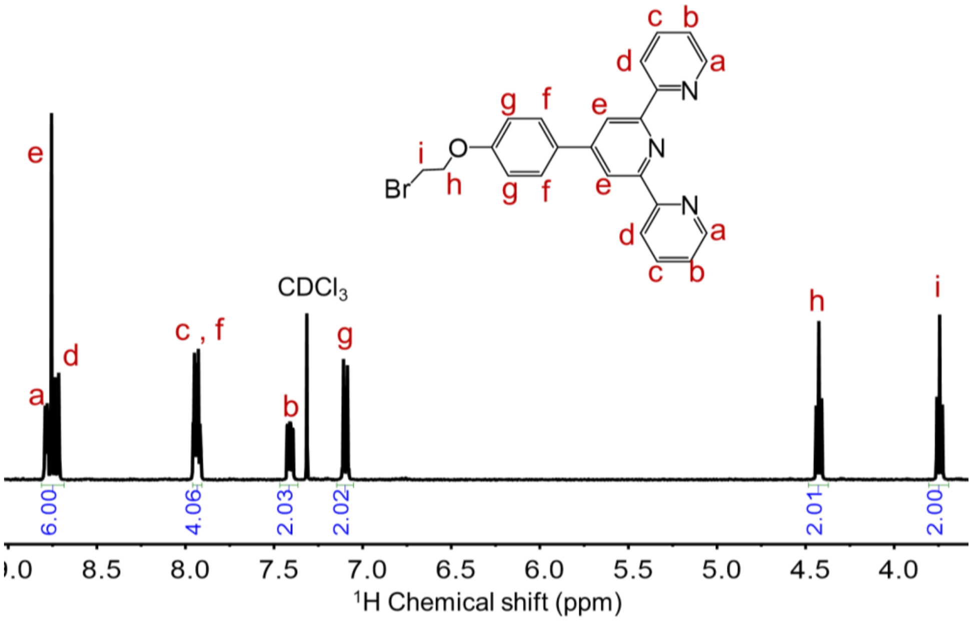

Figure S2. ^{1}H NMR spectrum ($CDCl_3$, 500 MHz, 298 K) of Br-TPY.

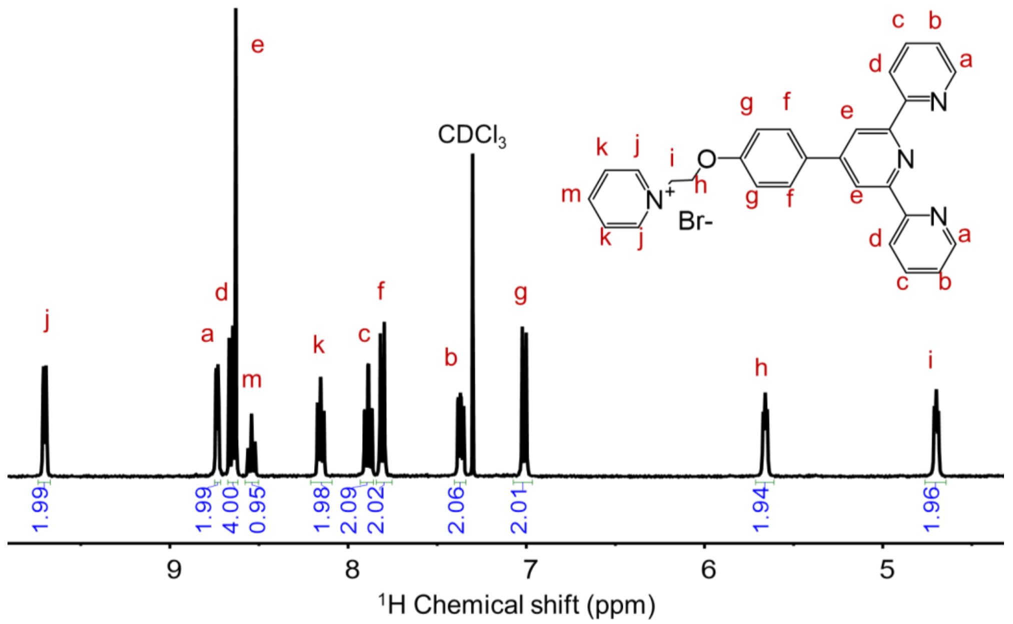

Figure S3. ^{1}H NMR spectrum ($CDCl_3$, 500 MHz, 298 K) of Py-TPY.

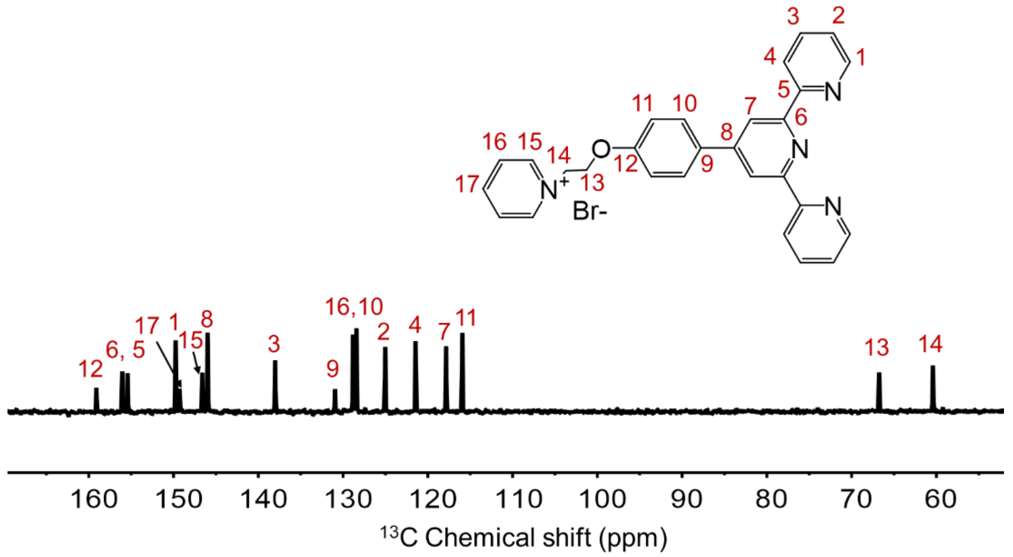

Figure S4. ^{13}C NMR spectrum ($CDCl_3$, 500 MHz, 298 K) of Py-TPY.

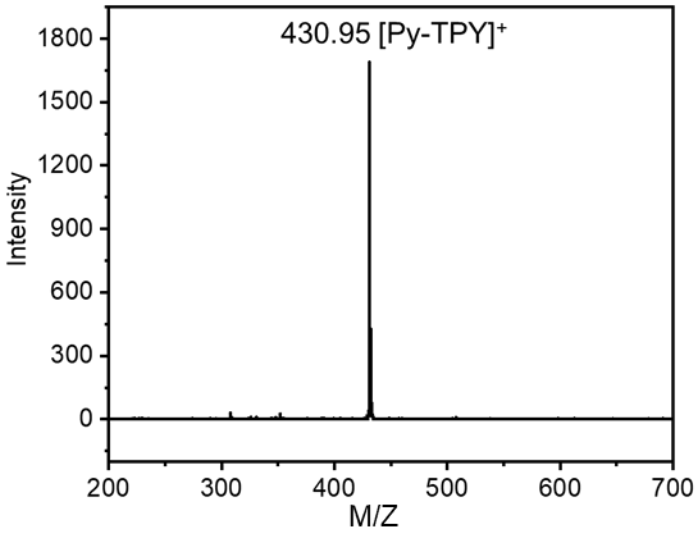

Figure S5. MALDI-TOF mass spectrum of Py-TPY.

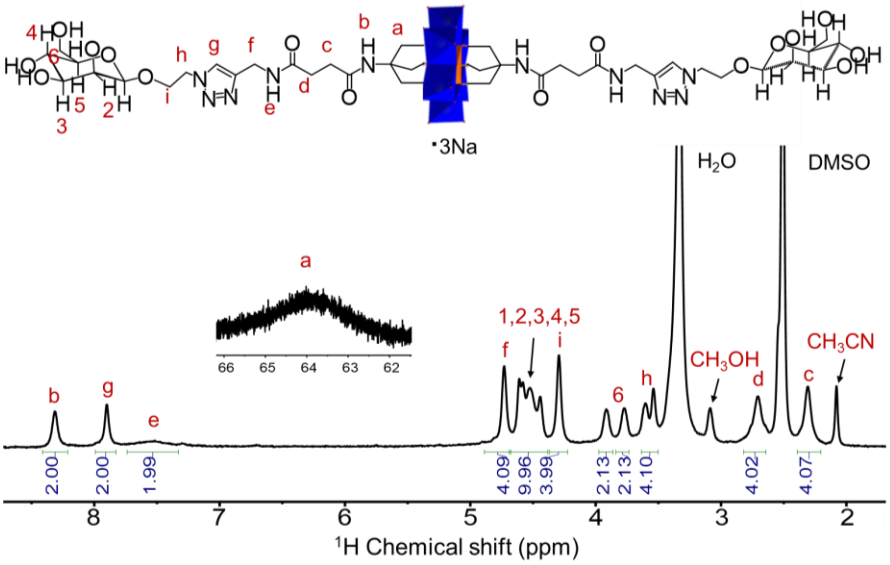

Figure S6. ^{1}H NMR spectrum (DMSO-d_6, 500 MHz, 298 K) of SMMM.

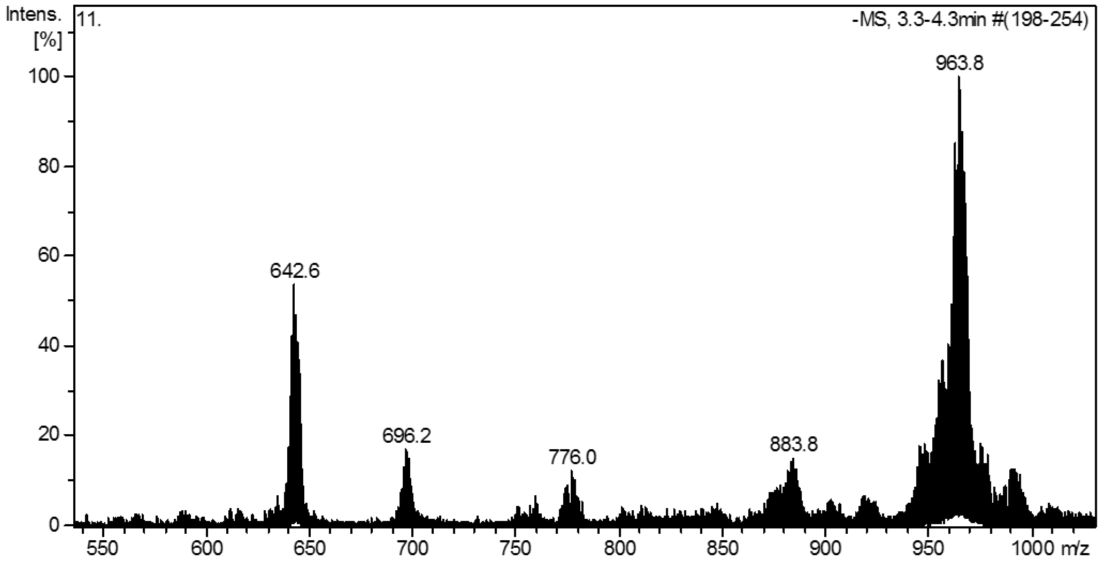

Figure S7. ESI-MS spectrum of SMMM.

Table S1. Assignment to the ESI-MS spectrum of SMMM.

Chemical formula	Charge	MW* Calculated	MW Found
$(H^+)[MnMo_6O_{24}](C_{38}H_{60}O_{16}N_{10})^{2-}$	$2e^-$	964.2	963.8
$[MnMo_6O_{24}](C_{38}H_{60}O_{16}N_{10})^{3-}$	$3e^-$	642.5	642.6

*MW: Molecular weight.

Table S2. Elemental analysis of SMMM.

SMMM	N(%)	C(%)	H(%)
Calculated	6.77	22.06	3.31
Found	6.54	21.92	3.58

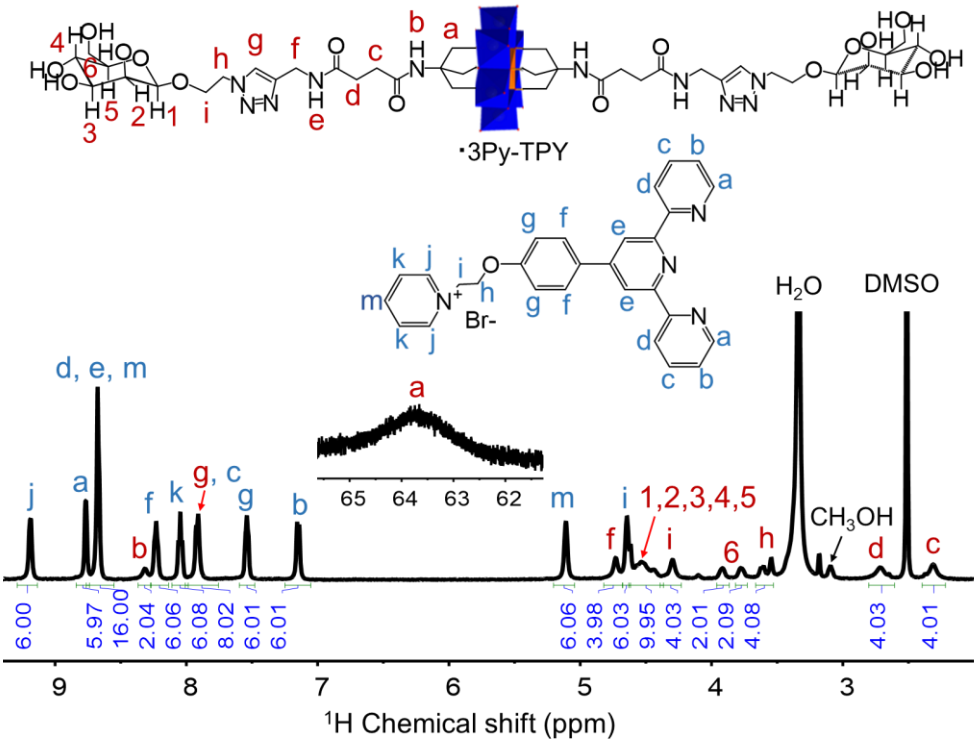

Figure S8. ^{1}H NMR spectrum (DMSO, 500 MHz, 298 K) of PMMM.

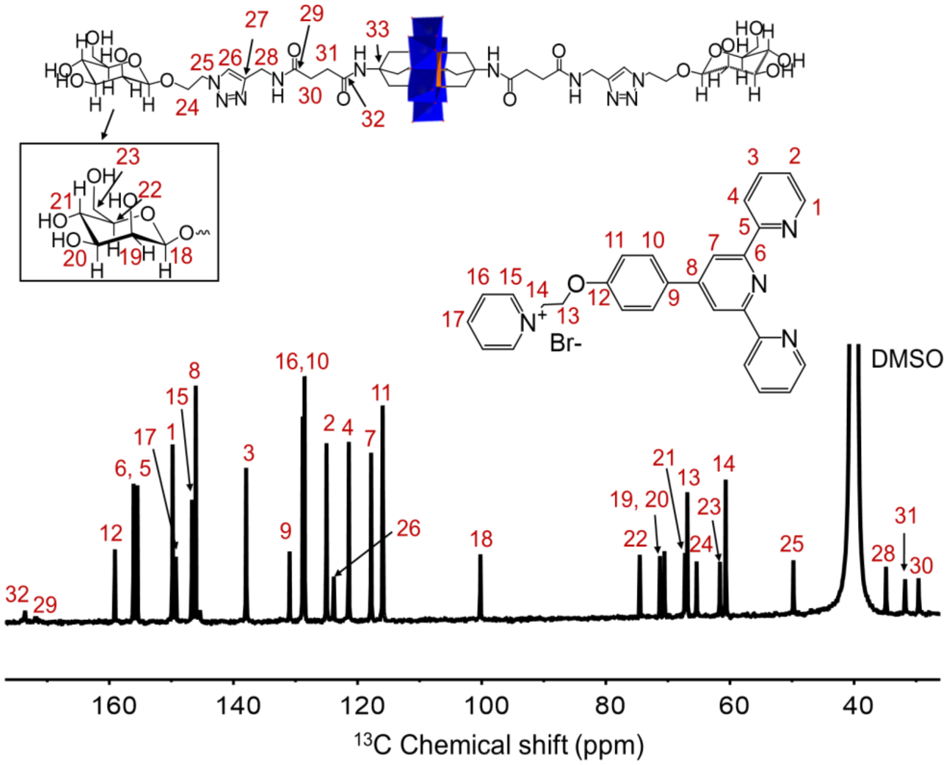

Figure S9. ^{13}C NMR spectrum (DMSO, 500 MHz, 298 K) of PMMM.

Table S3. Summary of ICP and organic elemental analysis of PMMM.

PMMM	N (%)	C (%)	H (%)	Mn (wt%)	Mo (wt%)
Calculated	8.89	42.40	4.53	1.65	17.38
Found	8.91	42.39	4.52	1.65	17.41

S5. Characterizations for metal coordination complexation

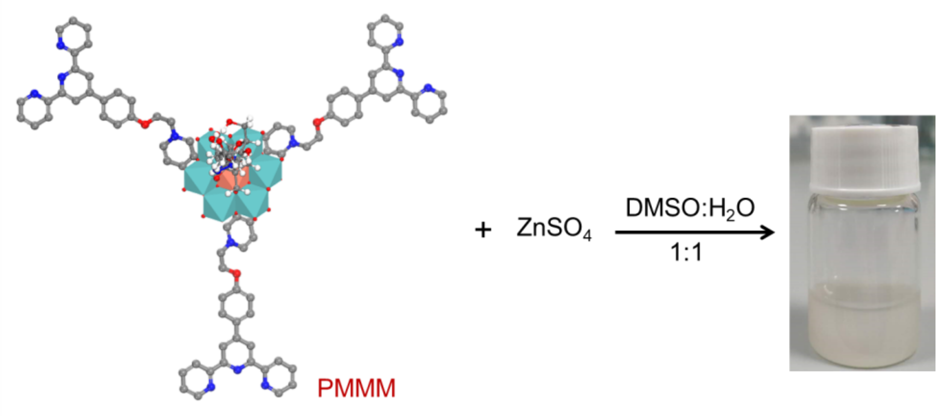

Figure S10. Self-assembly of PMMM (1.0 mg mL^{-1}) with $ZnSO_4$ at a molar ratio of 2:3 in $DMSO/H_2O$ (1:1 in volume ratio).

Table S4. The ICP and organic elemental analysis of PMMM-$ZnSO_4$ precipitate of 2D SF assembly.

PMMM-$ZnSO_4$	N (%)	C (%)	H (%)	S (%)	Mn (wt%)	Mo (wt%)	Zn (wt%)
Calculated	8.13	38.67	4.39	1.30	1.45	15.21	2.59
Found	7.84	38.84	4.02	1.23	1.35	15.54	2.51

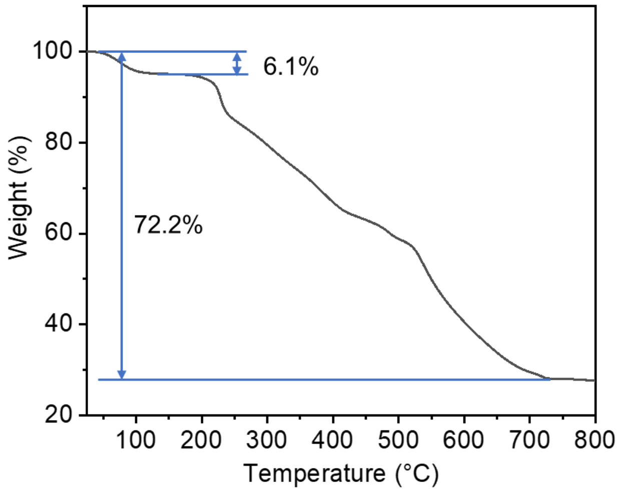

Figure S11. TGA curve of PMMM-$ZnSO_4$ precipitate of 2D SF assembly in $DMSO/H_2O$ (1:1 in volume ratio).

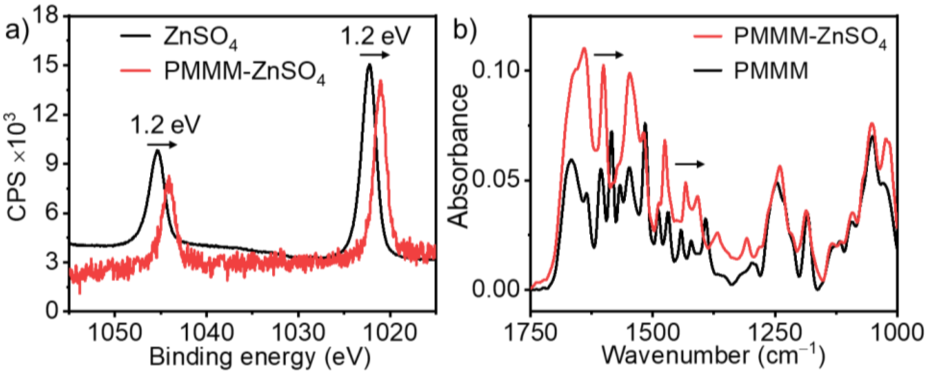

Figure S12. (a) XPS and (b) FT-IR spectra of $ZnSO_4$ and PMMM-$ZnSO_4$ 2D SF assembly ([PMMM]/[Zn^{2+}] = 1:1.5) in solid and KBr pellets.

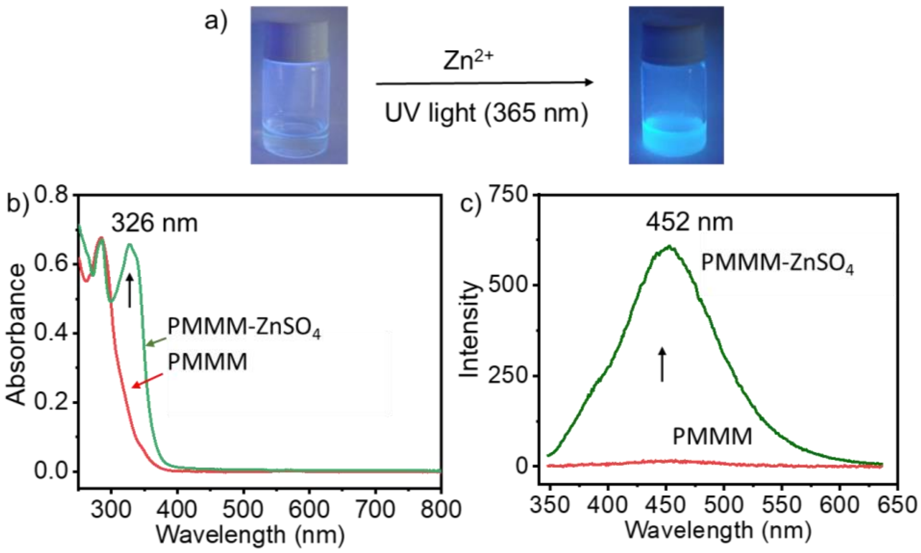

Figure S13. (a) Digital photographs of PMMM before and after adding Zn^{2+} (1.0 mg mL^{-1} in DMSO/H_2O at 1:1 in volume ratio) under the radiation of 365 nm light, and corresponding (b) UV-vis and (c) luminescent spectra at a concentration of 0.05 mg mL^{-1} in DMSO/H_2O (1:1 in volume ratio) at the excitation wavelength of 326 nm.

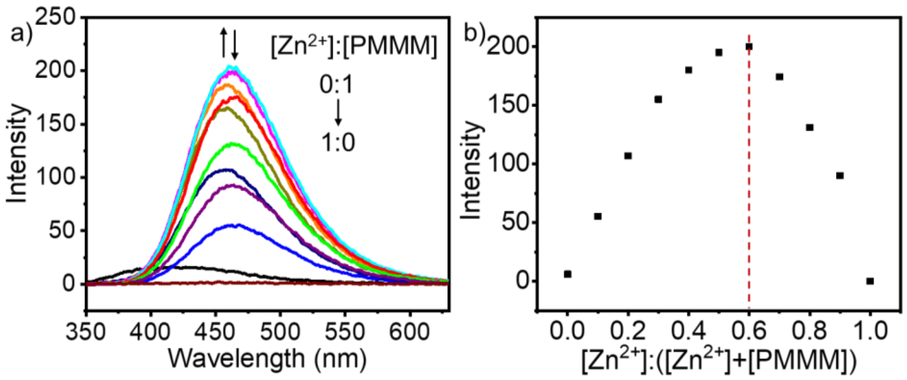

Figure S14. (a) Luminescence titration spectra for Zn^{2+} and PMMM versus the ratios of ([Zn^{2+}]:[PMMM] = 0:1, 1:9, 2:8, 3:7, 4:6, 5:5, 6:4, 7:3, 8:2, 9:1, 1:0) and (b) the corresponding Job's plot for Zn^{2+} and PMMM by monitoring the absorption at 452nm shown in (a).

S6. Characterizations for morphology and structures of framework assemblies.

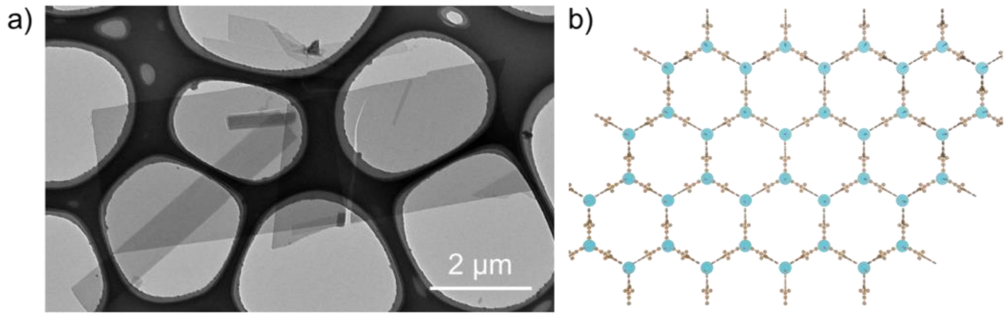

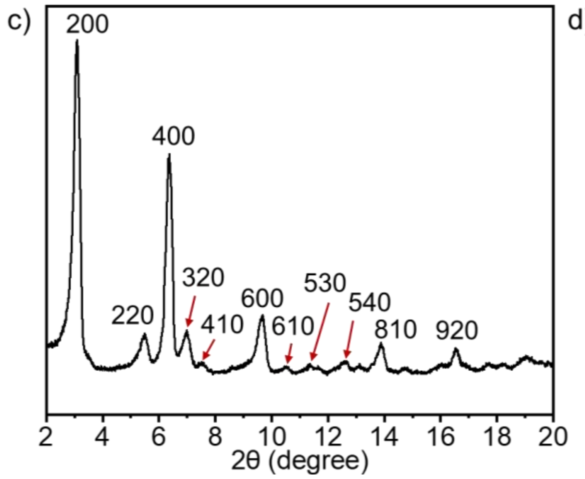

d)

2θ/°	d/nm	hkl
3.15	2.80	200
5.49	1.61	220
6.34	1.39	400
6.98	1.26	320
7.50	1.18	410
9.62	0.91	600
10.49	0.84	610
11.35	0.78	530
12.60	0.70	540
13.87	0.64	810
16.51	0.54	920

Figure S15. (a) TEM image of PMMM-Zn^{2+} 2D SF assembly prepared from its dispersion in DMSO/H_2O (1:1 in volume ratio) as a nanosheet, (b) simulation of the single-layer 2D SF assembly with a=6.4 nm, (c) powder XRD spectrum (same in Figure 2i) and (d) the corresponding peak indexation of the single-layer 2D SF solid.

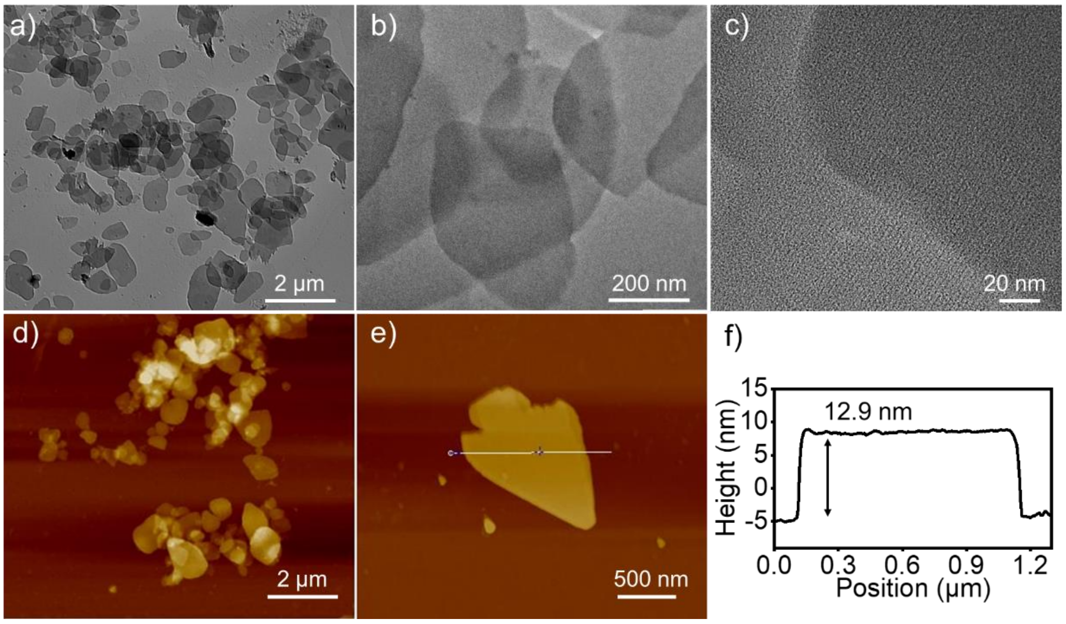

Figure S16. a, b, c) TEM and d, e) AFM images of multilayer 3D SF prepared from its assembly in DMSO/H_2O (1:1 in volume ratio) under sonication, in which the height profile analysis in f) is measured from e) in white line.

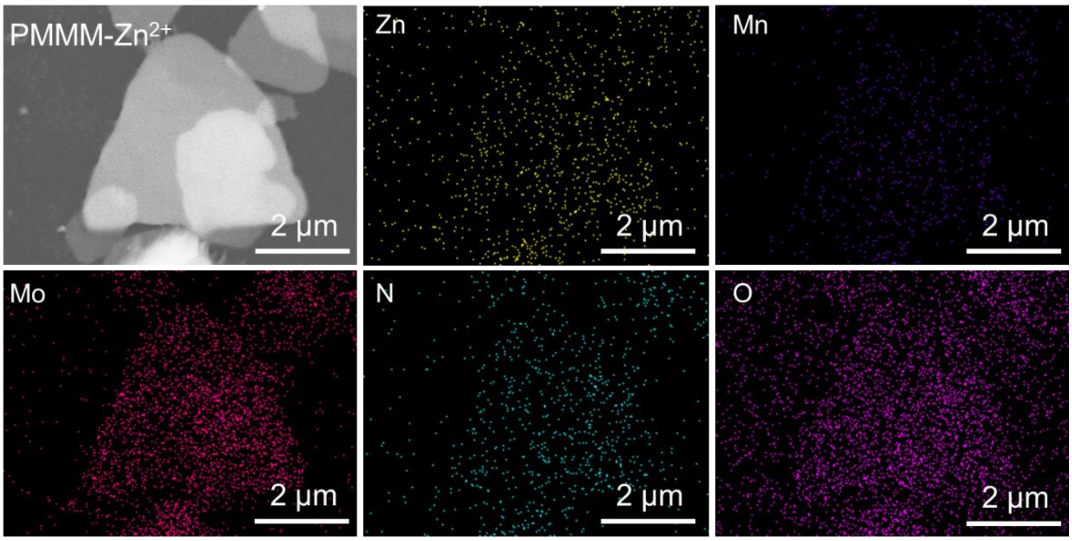

Figure S17. Element mapping images of nanosheets indicating the presence of zinc, manganese, and molybdenum elements in PMMM-Zn^{2+} multilayered 3D SF assembly.

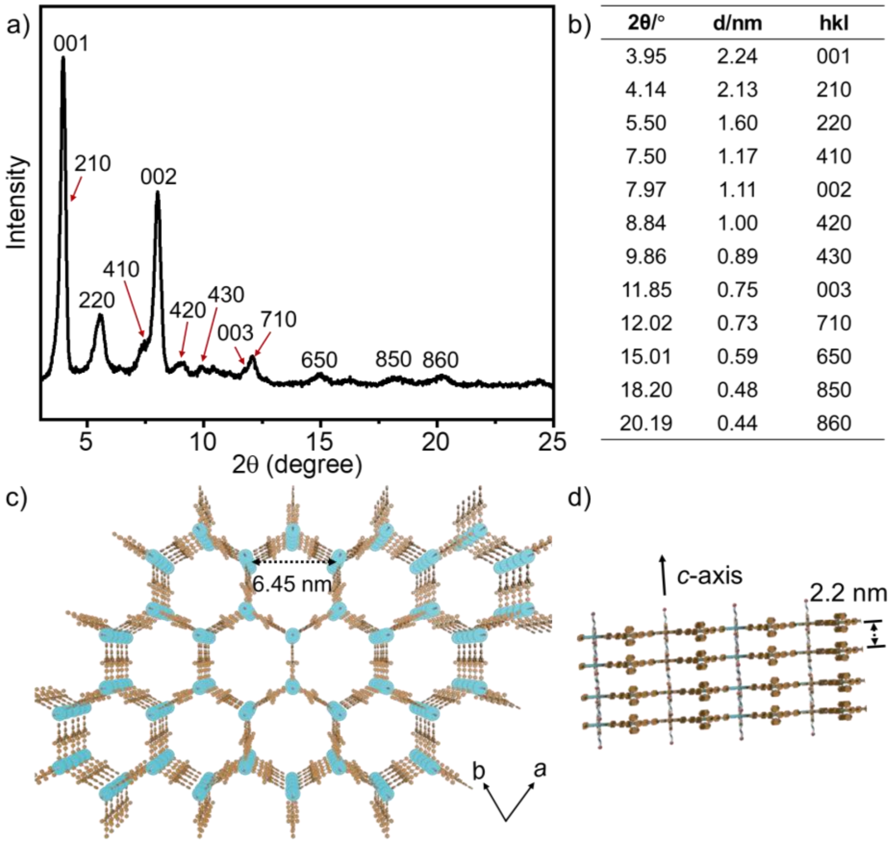

2θ/°	d/nm	hkl
3.95	2.24	001
4.14	2.13	210
5.50	1.60	220
7.50	1.17	410
7.97	1.11	002
8.84	1.00	420
9.86	0.89	430
11.85	0.75	003
12.02	0.73	710
15.01	0.59	650
18.20	0.48	850
20.19	0.44	860

Figure S18. (a) Powder XRD pattern and (b) peak indices of multilayer 3D SF assembly prepared from DMSO/H_2O (1:1 in volume ratio) under sonication, and simulation diagram of multilayer 3D SF at (c) ab and (d) c lattice plane.

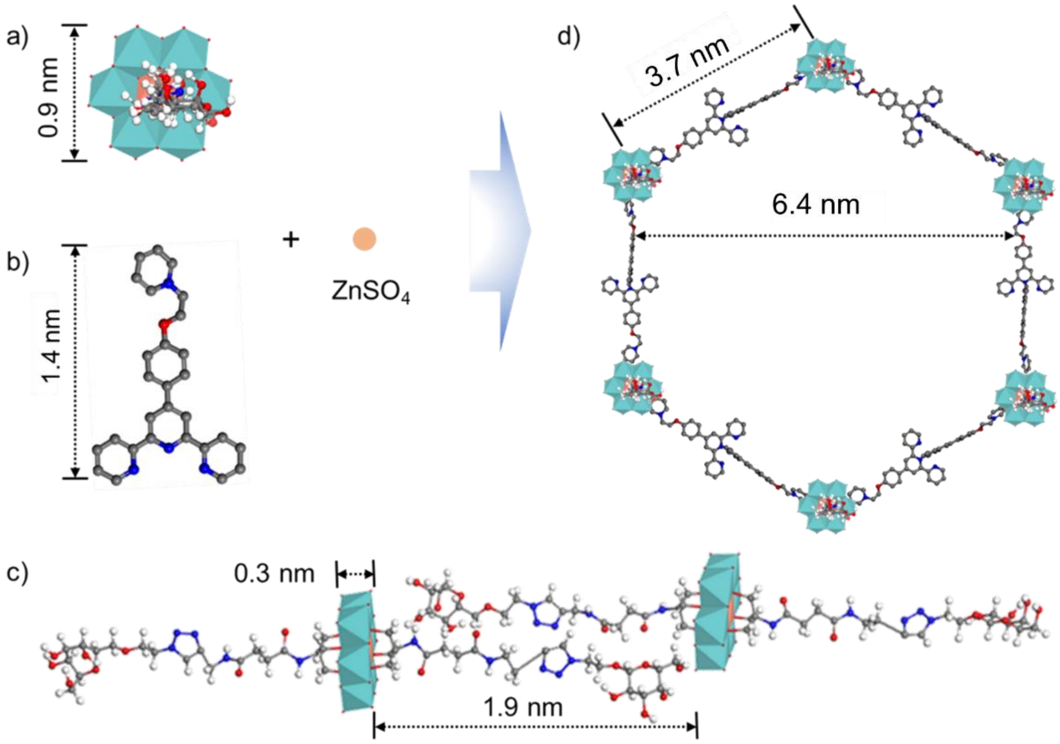

Figure S19. Structure model of (a) POM cluster MMM, (b) Py-TPY, (c) mannose group with spacer part, and (d) size fitting to the proposed framework.

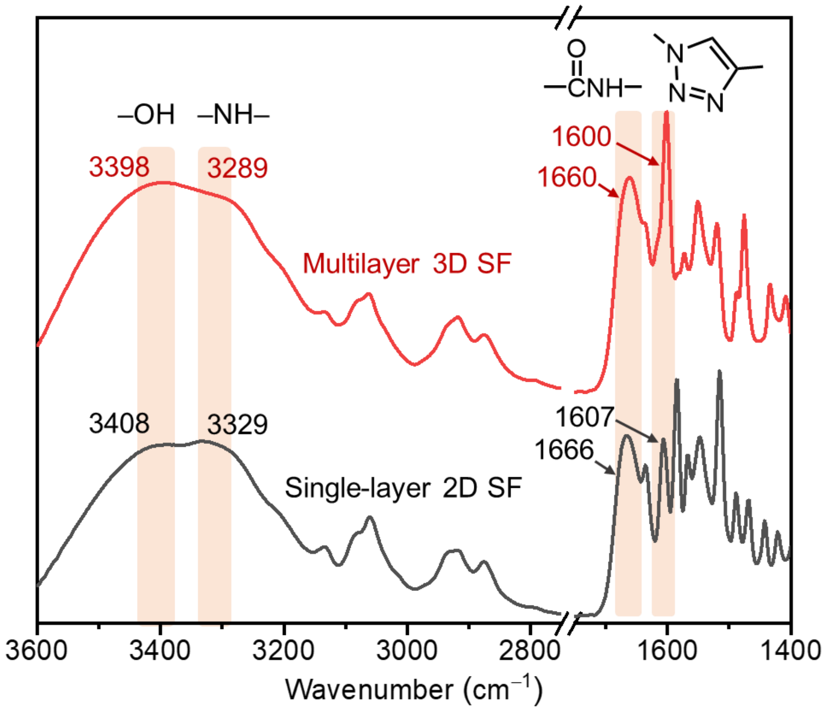

Figure S20. FT-IR spectra of single layer 2D SF and multilayer 3D SF in KBr pellet with assigned peaks belonging to amide, hydroxy and triazole groups.

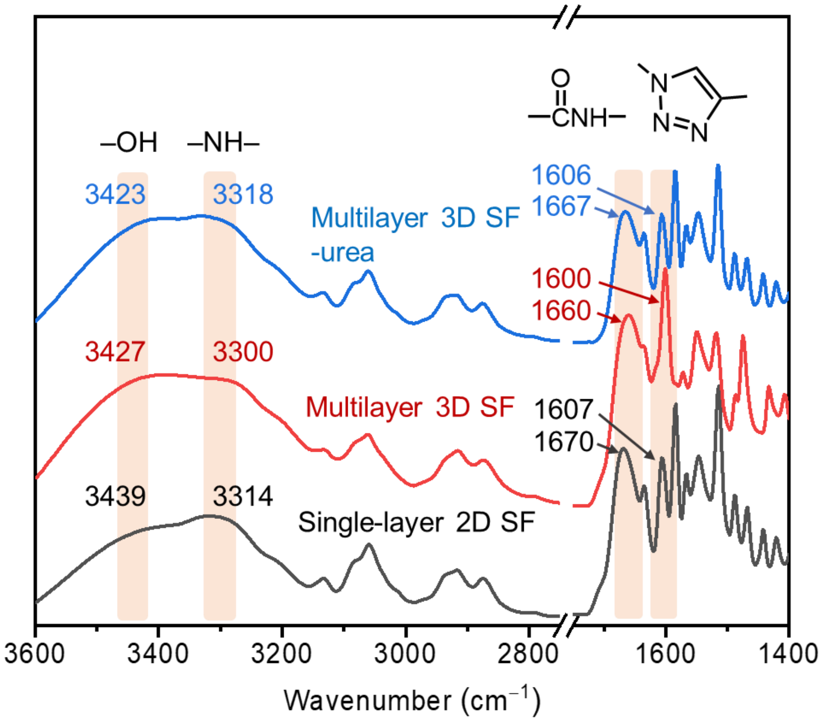

Figure S21. FT-IR spectra of single layer 2D SF, multilayer 3D SF and multilayer 3D SF with excess urea in KBr for the same batch of measurement, in which the assigned vibration bands belonging to amide, hydroxy and triazole groups.

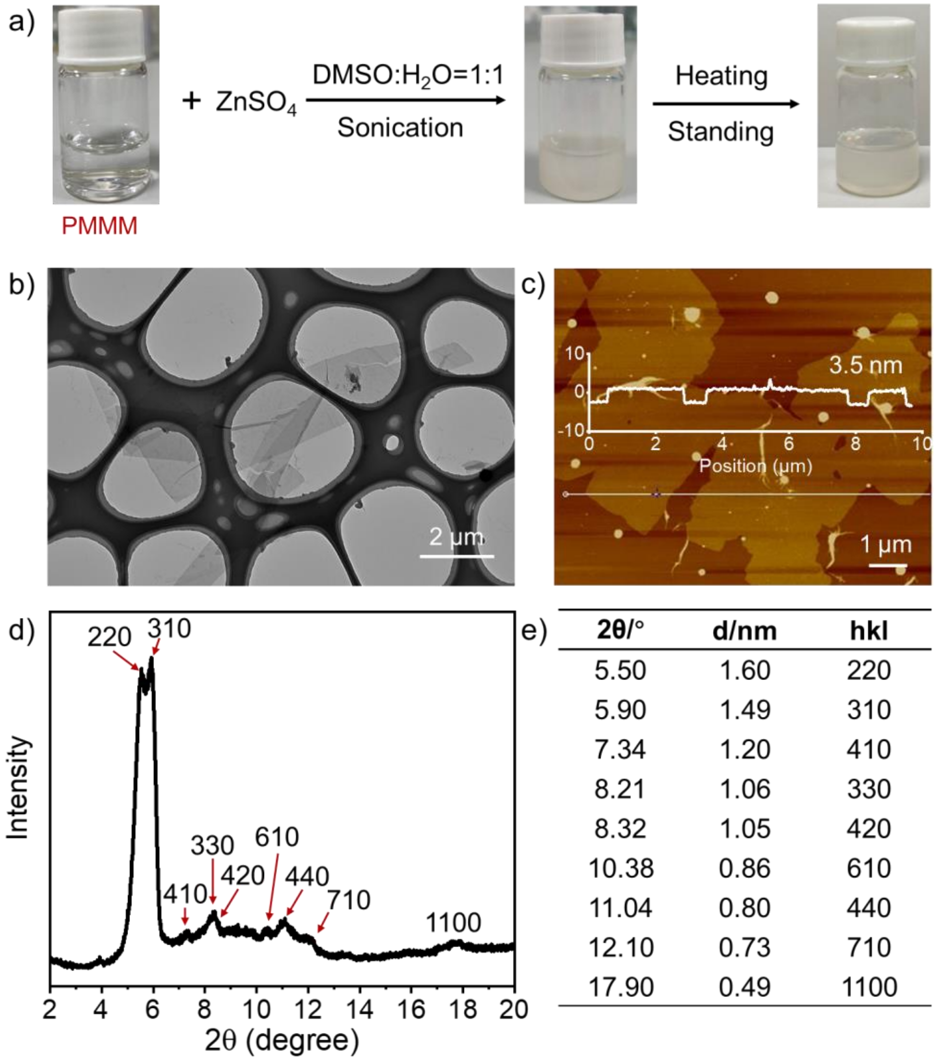

2θ/°	d/nm	hkl
5.50	1.60	220
5.90	1.49	310
7.34	1.20	410
8.21	1.06	330
8.32	1.05	420
10.38	0.86	610
11.04	0.80	440
12.10	0.73	710
17.90	0.49	1100

Figure S22. (a) Digital photograph of single layer 2D SF assembly solution after sonication in DMSO/H_2O (1:1 in volume ratio), (b) TEM and (c) AFM images of single layer nanosheets, (d) powder XRD pattern and (e) peak indices of the solid of the single-layer 2D SF assembly.

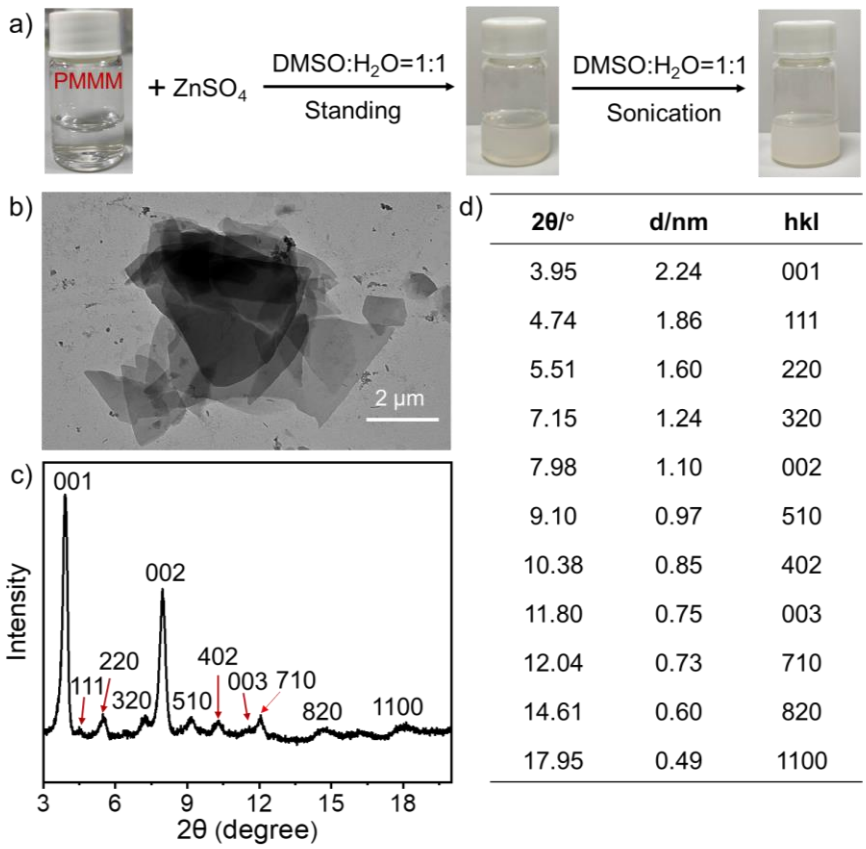

2θ/°	d/nm	hkl
3.95	2.24	001
4.74	1.86	111
5.51	1.60	220
7.15	1.24	320
7.98	1.10	002
9.10	0.97	510
10.38	0.85	402
11.80	0.75	003
12.04	0.73	710
14.61	0.60	820
17.95	0.49	1100

Figure S23. (a) Digital photographs of the preparation of multilayer 3D SF assembly by sonication in DMSO/H_2O (1:1 in volume ratio), (b) TEM image of the multilayer nanosheets, (c) powder XRD pattern and (d) corresponding peak indices of multilayer 3D SF assembly in solid.

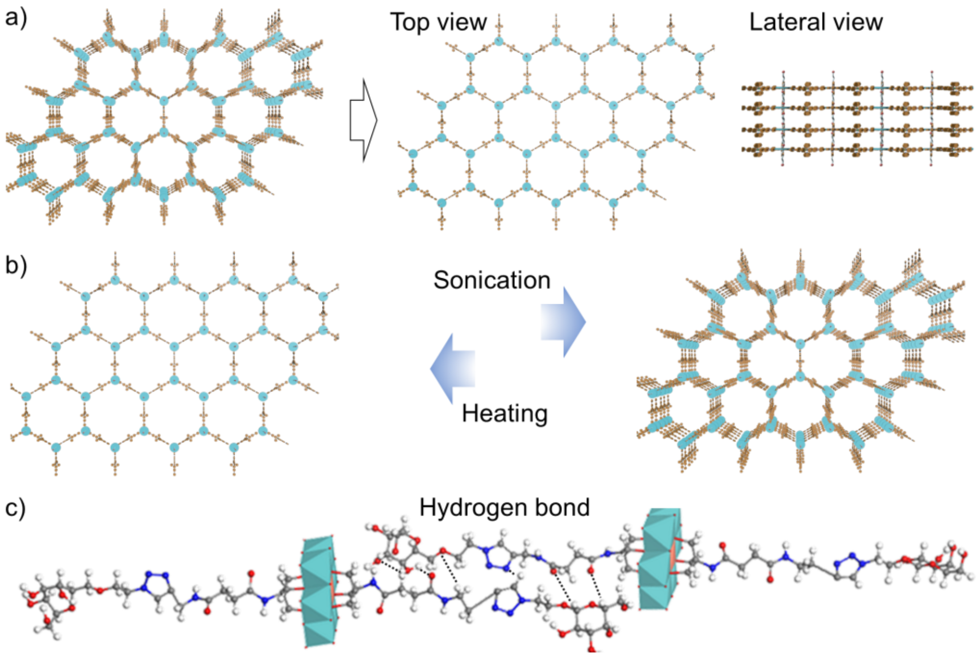

Figure S24. (a) Simulation structure of multilayer 3D SF from top view and lateral view, (b) interconversion between single layer and multilayer 3D SF, and (c) possible model of interlayer hydrogen bond that suffers from the sonication and heating.

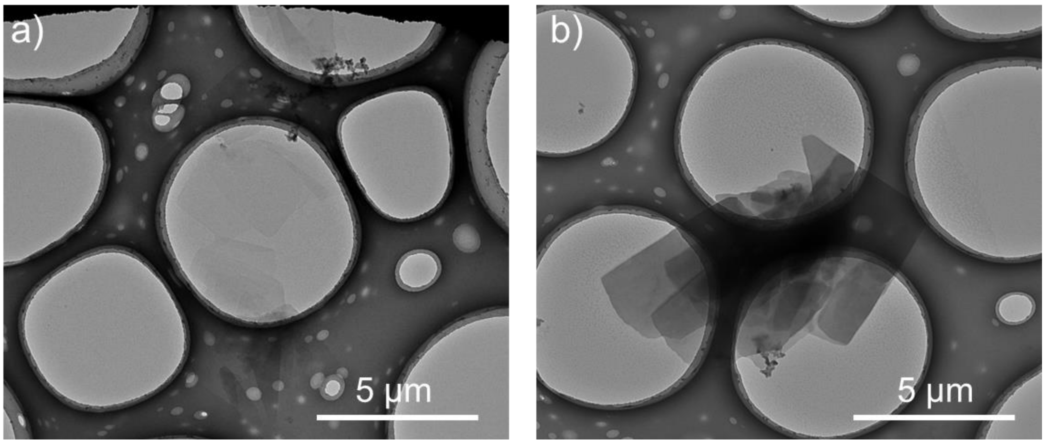

Figure S25. TEM images of the initial formed single layer nanosheets after (a) 2 h and (b) 12 h of aging in DMSO/H_2O.

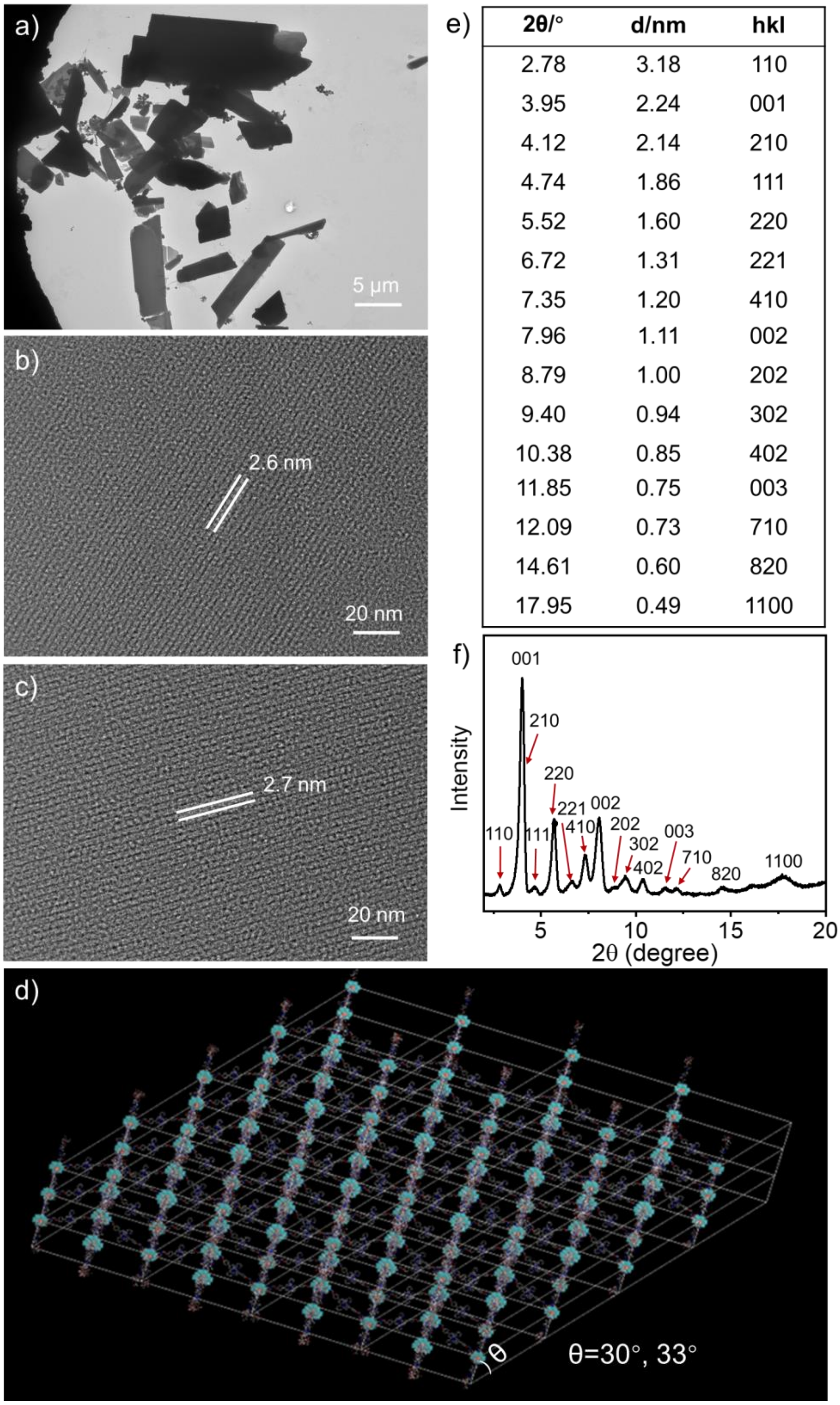

2θ/°	d/nm	hkl
2.78	3.18	110
3.95	2.24	001
4.12	2.14	210
4.74	1.86	111
5.52	1.60	220
6.72	1.31	221
7.35	1.20	410
7.96	1.11	002
8.79	1.00	202
9.40	0.94	302
10.38	0.85	402
11.85	0.75	003
12.09	0.73	710
14.61	0.60	820
17.95	0.49	1100

Figure S26. TEM images of granular 3D SF assemblies prepared from dispersions in water (a) at large scale and magnification which present lattice fringes from the calculated angle of (b) 2θ 32° and (c) 2θ 35°, (d) the simulation diagram of (110) at different angles, (e) powder XRD pattern and (f) corresponding peak indices of the dispersed granular 3D SF solid.

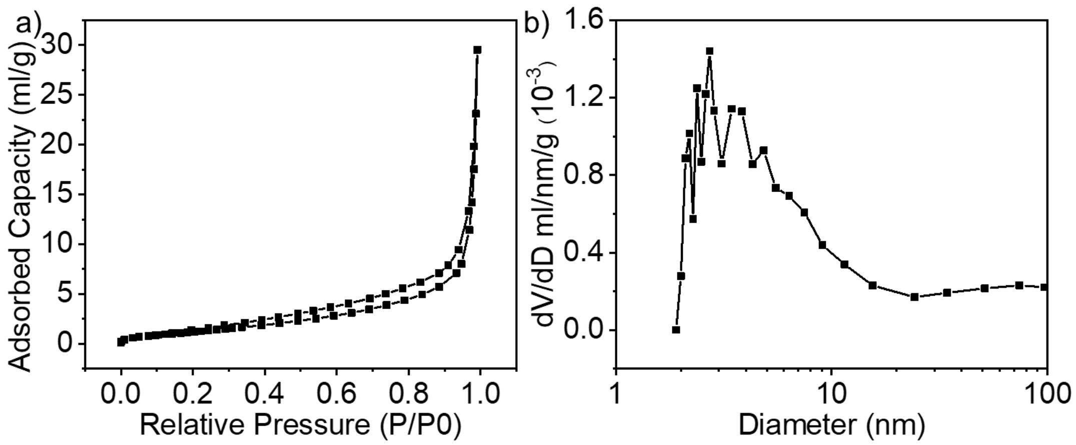

Figure S27. (a) Nitrogen sorption isotherm at 77 K and (b) calculated pore size distributions of granular 3D SF.

S7. Size-selective separation of gold nanoparticles.

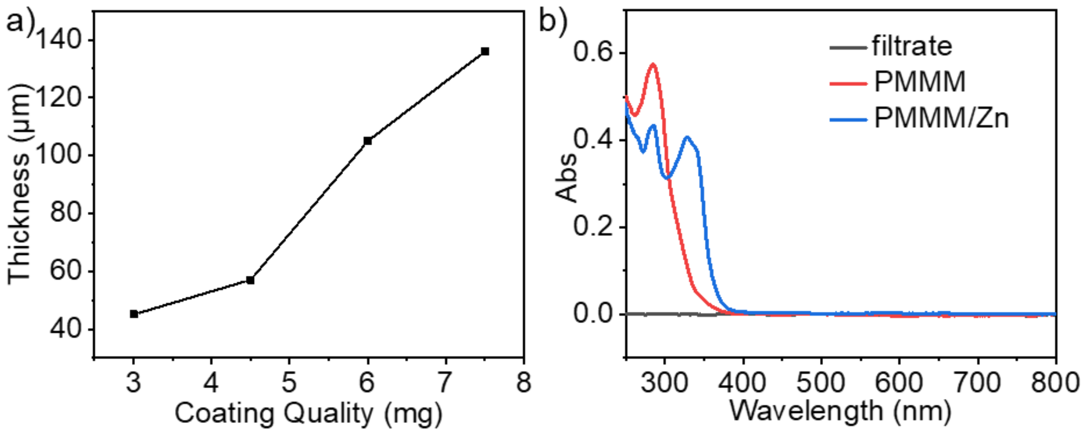

Figure S28. (a) thickness plot of membrane versus casting quality of sample dispersion (0.1 mg mL^{-1}) in DMSO/H_2O (1:1 v/v), and (b) UV-vis spectra of the filtrate from membrane and the isolated components in DMSO.

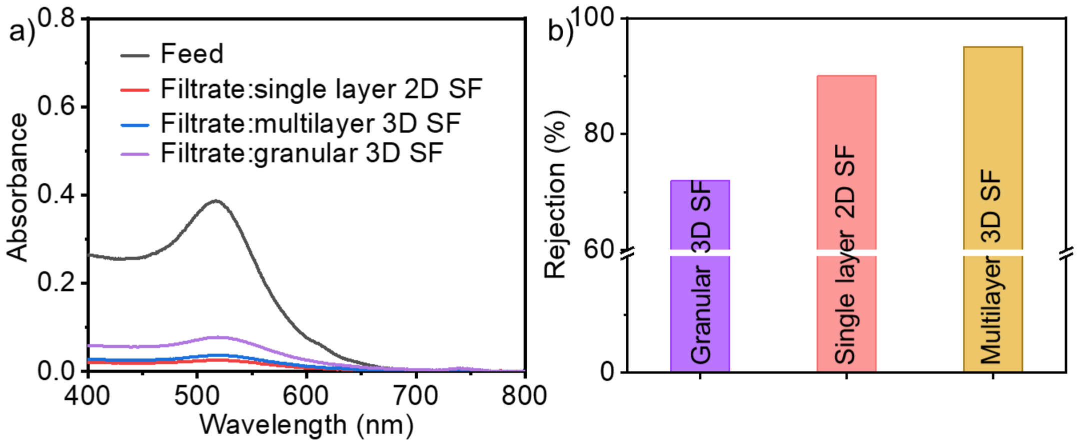

Figure S29. (a) UV-vis spectra of AuNPs (9 nm) aqueous solution before and after filtration through the membrane prepared from different assembly conditions under reduced pressure and (b) corresponding histogram of rejection efficiency.

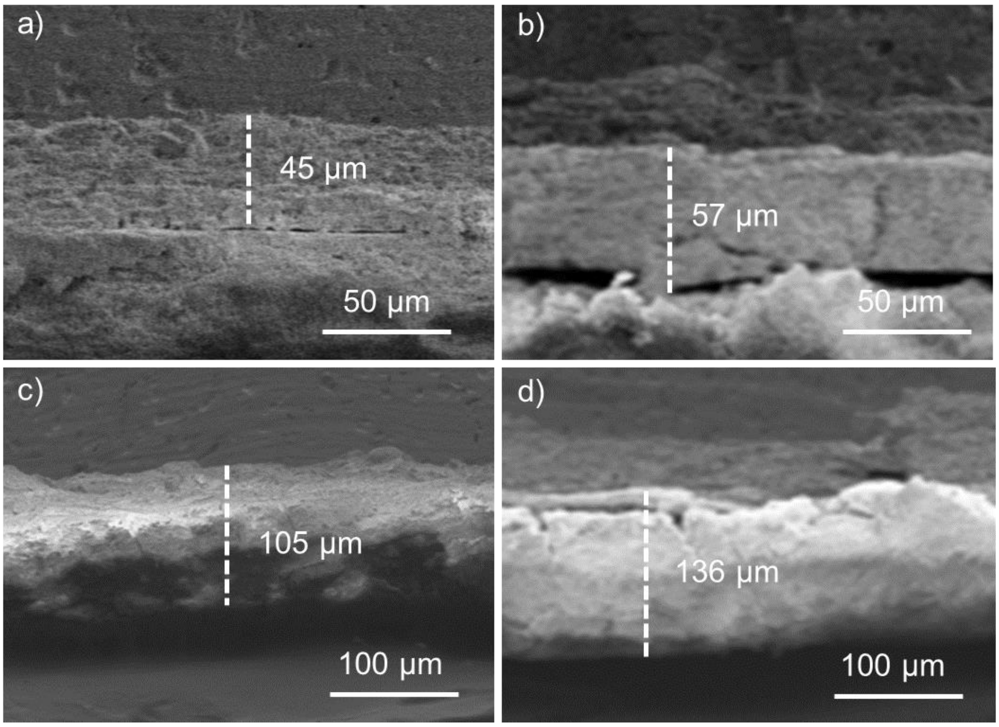

Figure S30. Cross-sectional SEM images of membranes with coating volume of (a) 1.0 mL, (b) 1.5 mL, (c) 2.0 mL, and (d) 2.5 mL of multilayer 3D SF in DMSO/H_2O (1:1 in volume ratio) mixed solution (3 mg mL^{-1}).

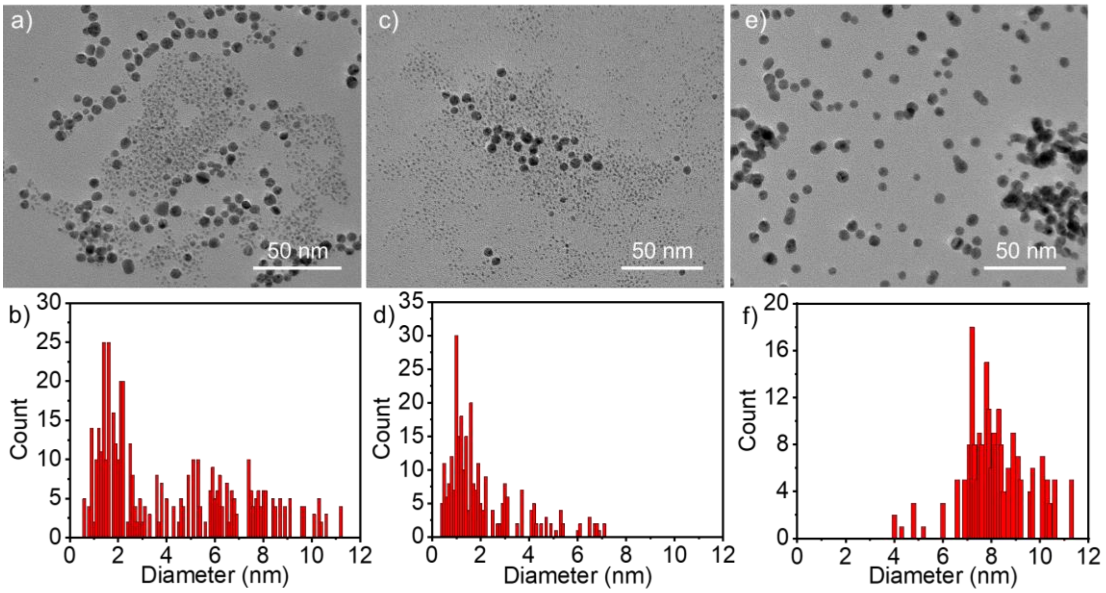

Figure S31. TEM images and corresponding statistic histograms (particle size) of Au NPs (mixture size of 0.5−11.0 nm) from (a, b) feed solution, (c, d) filtrate, and (e, f) retentate solution after the filtration of membrane M45.

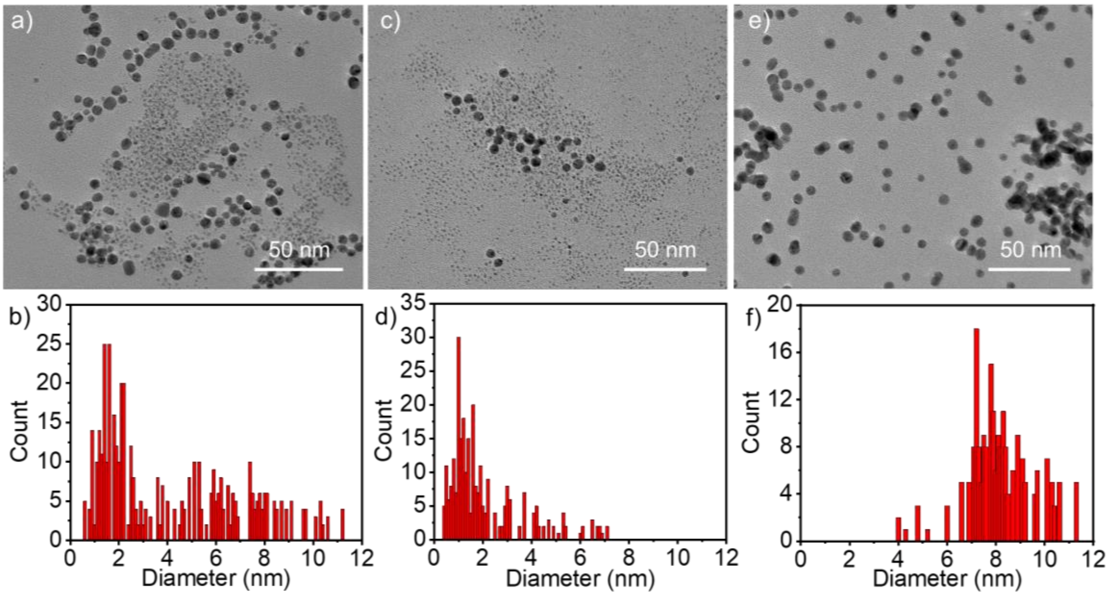

Figure S32. TEM images and corresponding statistic histograms (particle size) of Au NPs (mixture size of 0.5−11.0 nm) from (a, b) feed solution, (c, d) filtrate, and (e, f) retentate solution after the filtration of membrane M57.

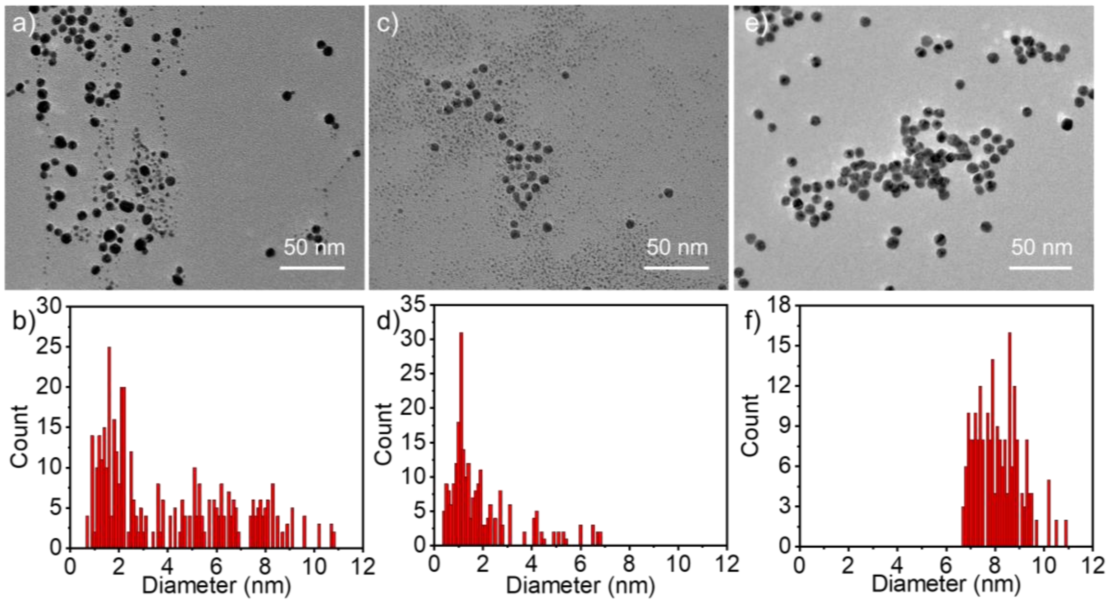

Figure S33. TEM images and corresponding statistic histograms (particle size) of Au NPs (mixture size of 0.5−11.0 nm) from (a, b) feed solution, (c, d) filtrate, and (e, f) retentate solution after the filtration of membrane M105.

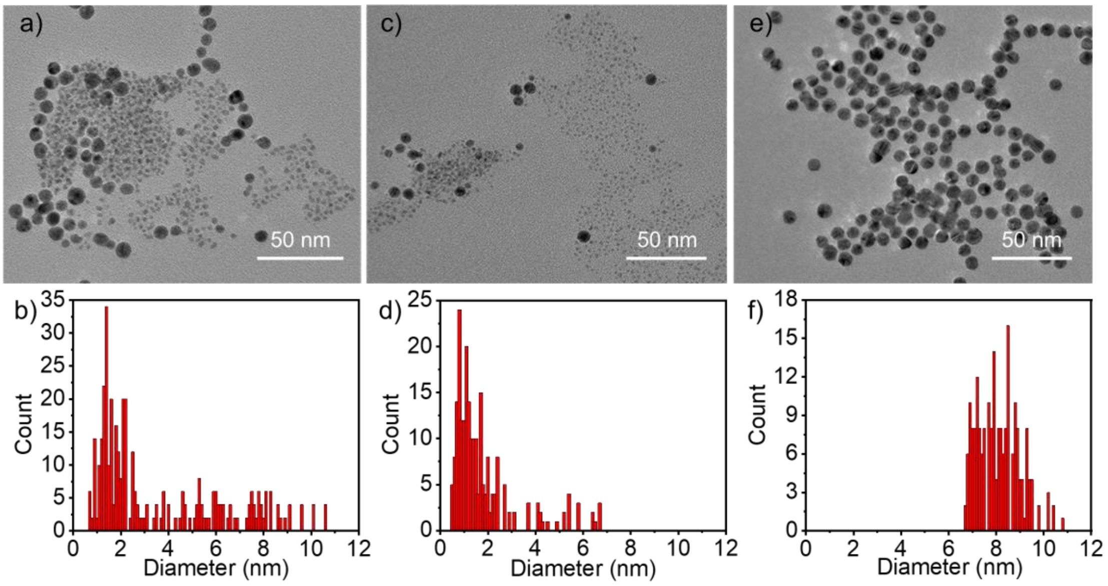

Figure S34. TEM images and corresponding statistic histograms (particle size) of Au NPs (mixture size of 0.5−11.0 nm) from (a, b) feed solution, (c, d) filtrate, and (e, f) retentate solution after the filtration of membrane M136.

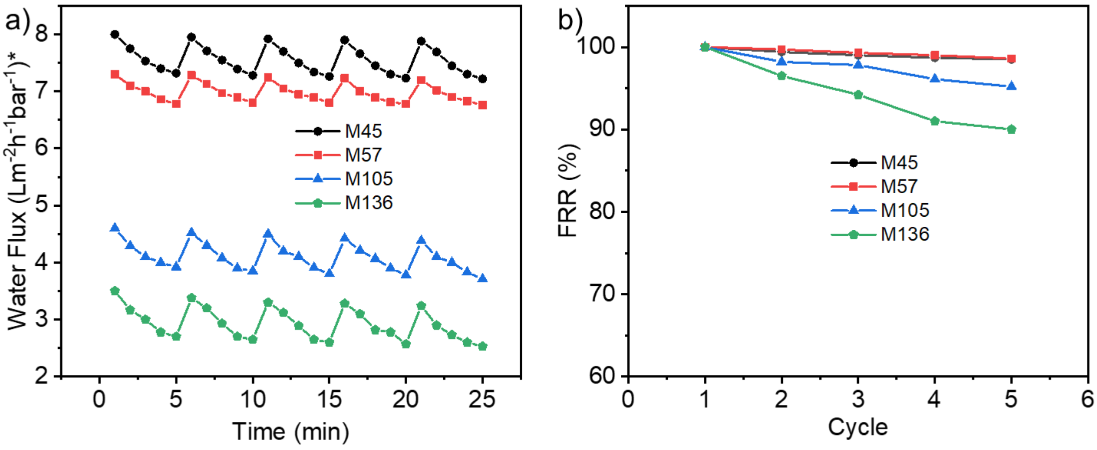

Figure S35. (a) Plots of water flux (in LMH) versus time for the membranes with different thicknesses under a pressure of -0.08 MPa, and (b) recovery rate of flux (FRR) at different cycles, in which “*” means the flux value×1000.

S8. Size-selective separation of proteins

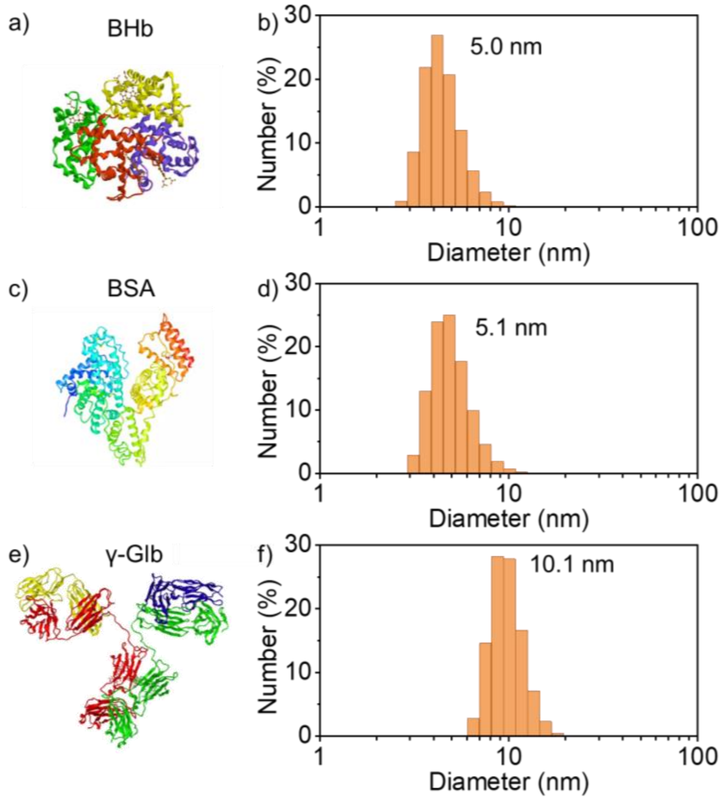

Figure S36. Crystal structures and DLS particle size distributions of (a, b) BHb, (c, d) BSA, and (e, f) γ-Glb.

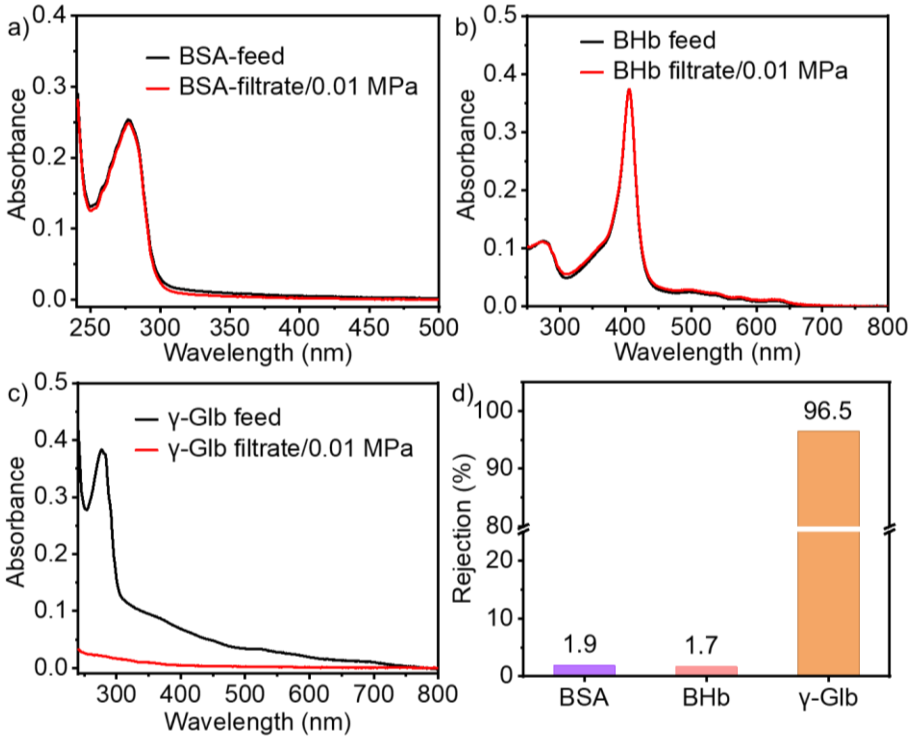

Figure S37. UV-vis spectra of (a) BSA, (b) BHb and (c) γ-Glb aqueous solution before and after filtration through the membrane M136, and (d) corresponding histogram of rejection efficiency.

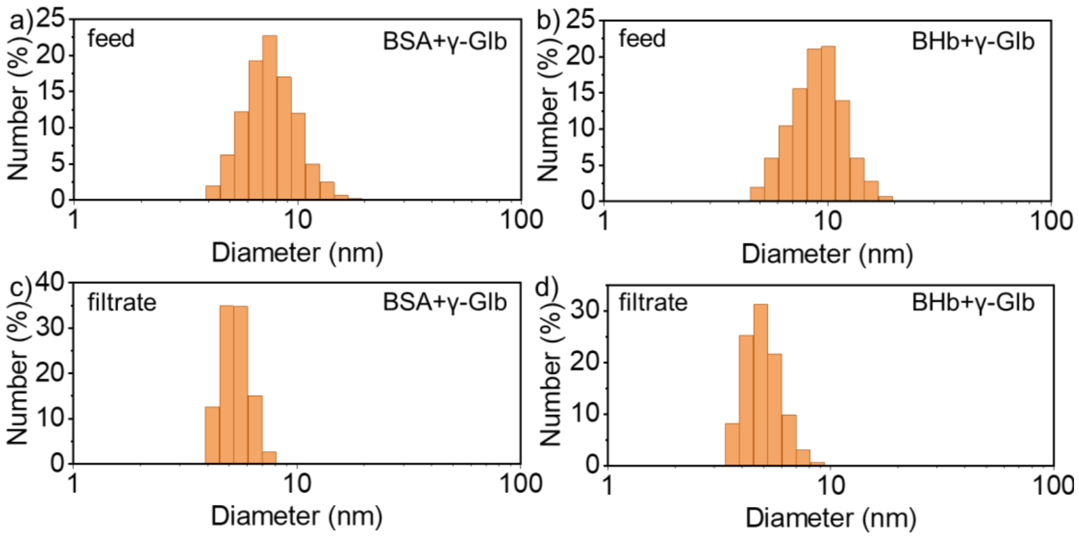

Figure S38. DLS plots of BSA and BHb in their mixture aqueous solutions with γ-Glb (a, b) before and (c, d) after filtration through the membrane M136 (C_{BSA}:$C_{\gamma\text{-Glb}}$=2:1 and C_{BHb}:$C_{\gamma\text{-Glb}}$=1:2).

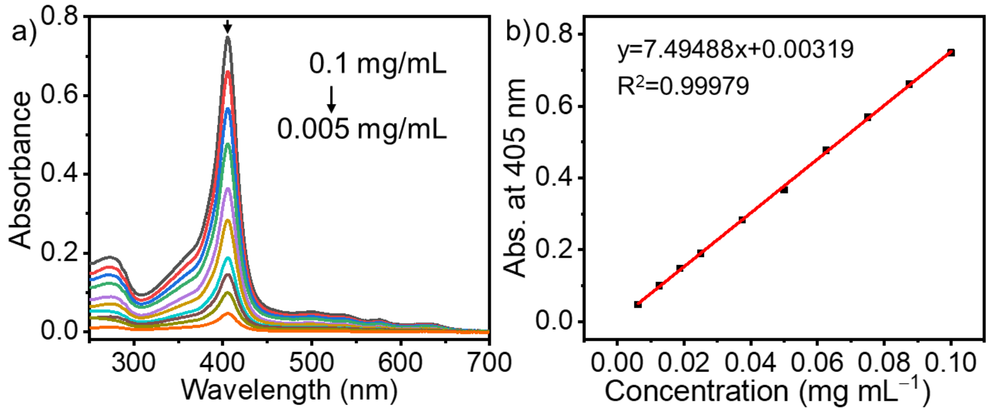

Figure S39. (a) UV-vis spectra of BHb in water at the concentrations of 0.1, 0.09, 0.075, 0.06, 0.05, 0.035, 0.025, 0.02, 0.0125, and 0.005 mg mL^{-1} from top to bottom, and (b) corresponding plot of absorbance at 405 nm upon the concentration increase.

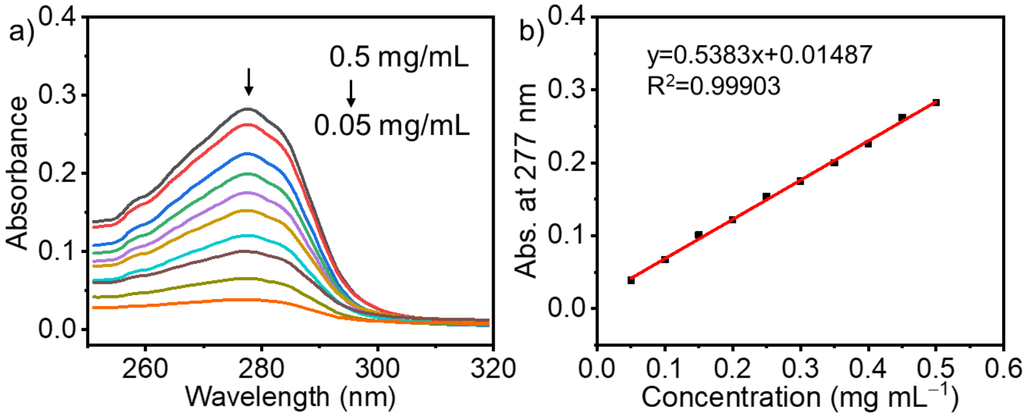

Figure S40. (a) UV-vis spectra of BSA in water at the concentrations of 0.5, 0.45, 0.4, 0.35, 0.3, 0.25, 0.2, 0.15, 0.1, and 0.05 mg mL^{-1} from top to bottom, and (b) corresponding plot of absorbance at 277 nm upon the concentration increase.

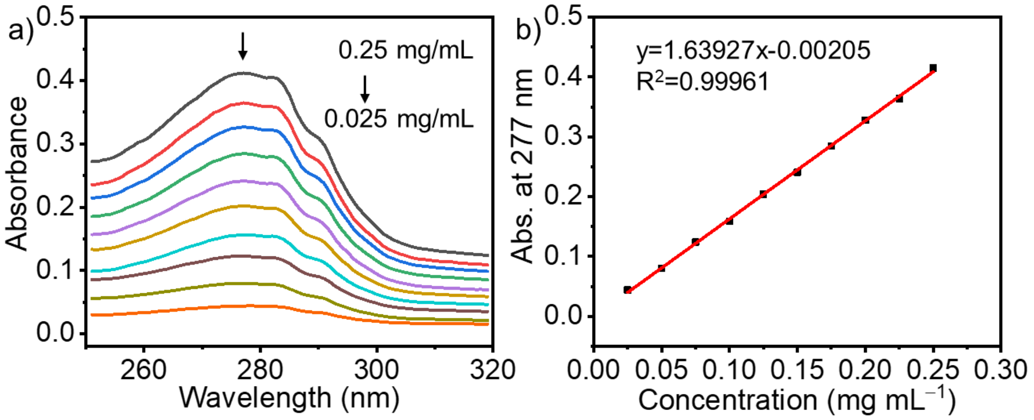

Figure S41. (a) UV-vis spectra of γ-Glb in water at the concentration of 0.25, 0.225, 0.2, 0.175, 0.15, 0.125, 0.1, 0.075, 0.05, and 0.025 mg mL^{-1} from top to bottom, and (b) corresponding plot of absorbance change at 277 nm upon the concentration increase.

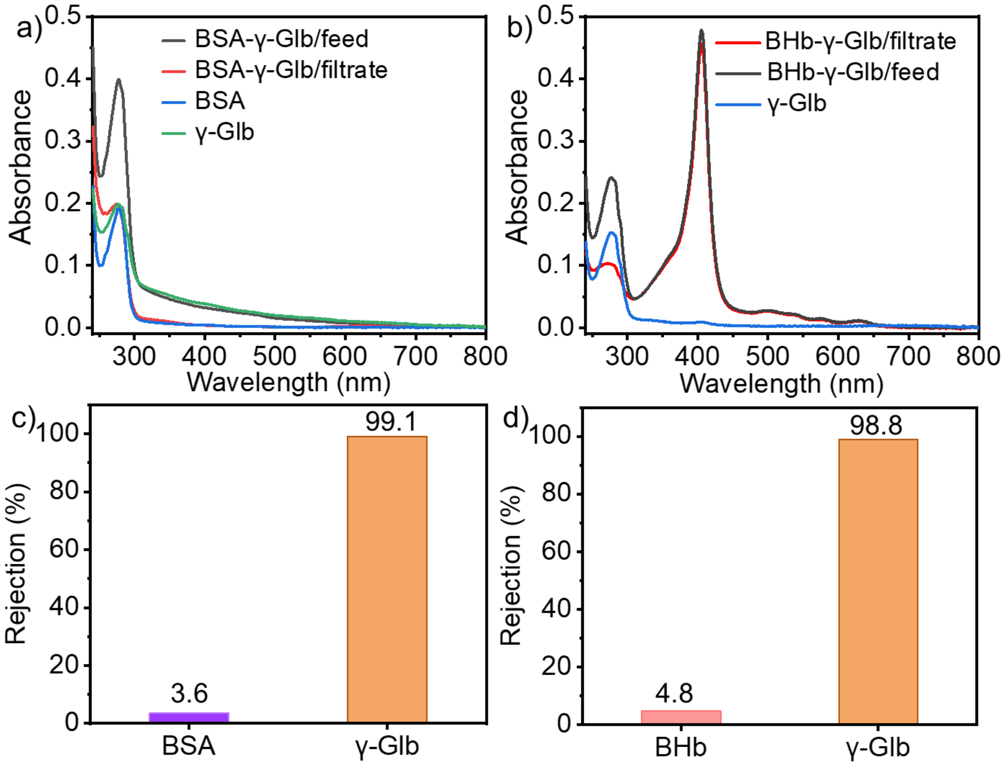

Figure S42. UV-vis spectra of (a) BSA and (b) BHb in the mixture aqueous solutions with γ-Glb before and after filtration through membrane M136 with the rejection histograms for the mixture of (c) BSA and γ-Glb and (d) BHb and γ-Glb.

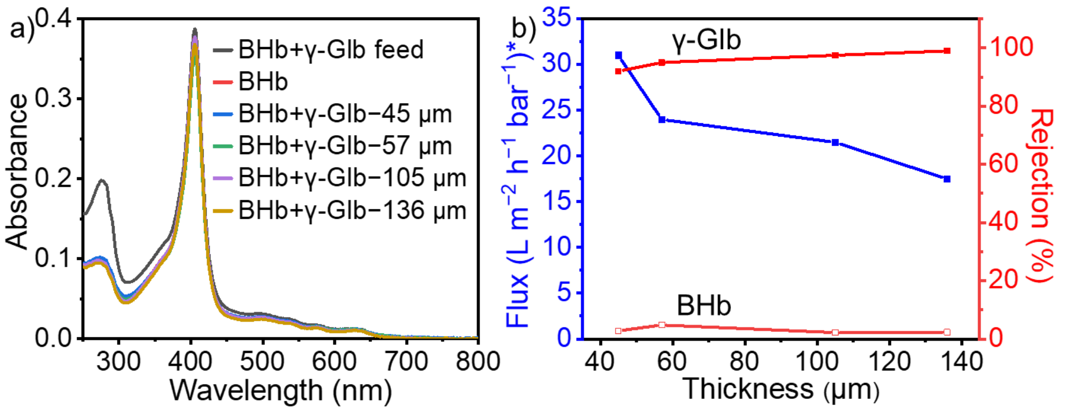

Figure S43. (a) UV-vis spectra of mixture aqueous solutions of BHb and γ-Glb before and after separation of membranes with different thicknesses at −0.01 MPa, and (b) plots of water flux and rejection efficiency versus the change of membrane thickness. "*" means the flux value×100.

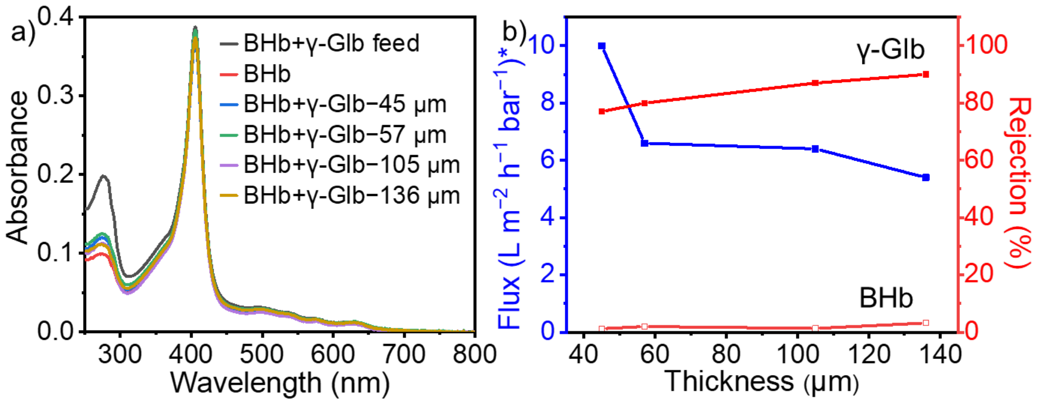

Figure S44. (a) UV-vis spectra of mixture aqueous solutions of BHb and γ-Glb before and after separation via the membranes with different thicknesses at −0.05 MPa, and (b) plots of water flux and rejection efficiency versus the membrane thickness. "*" means the flux value×100.

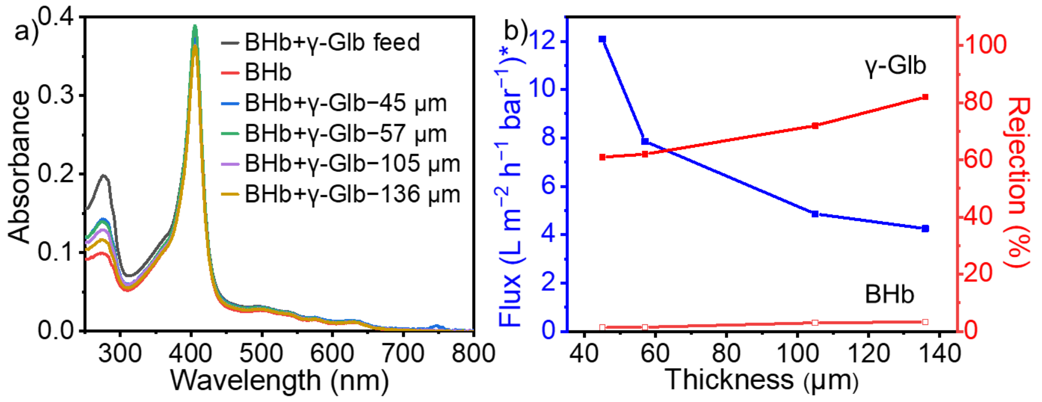

Figure S45. (a) UV-vis spectra of mixture aqueous solutions of BHb and γ-Glb before and after separation via the membranes with different thicknesses at −0.09 MPa, and (b) plots of water flux and rejection efficiency versus the membrane thickness. "*" means the flux value×100.

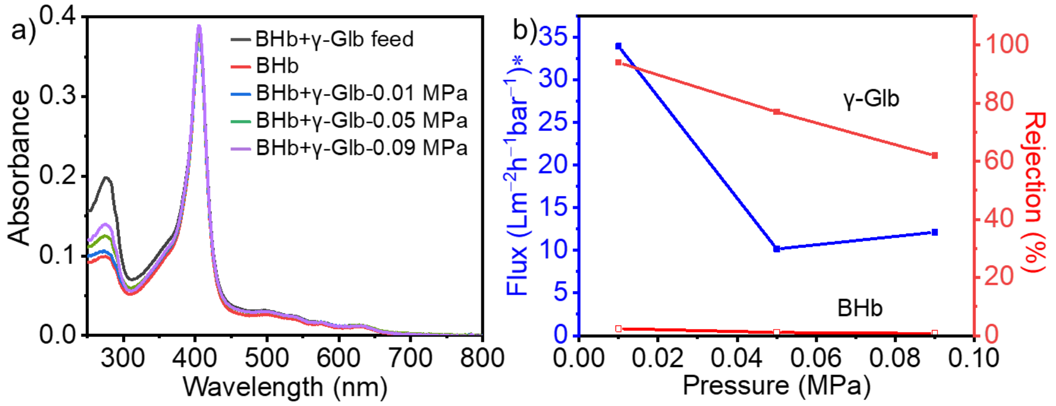

Figure S46. (a) UV-vis spectra of BHb and γ-Glb mixture aqueous solutions before and after separation through membrane M45, and (b) plots of water flux and rejection efficiency versus the change of pressure. "*" means the flux value×100.

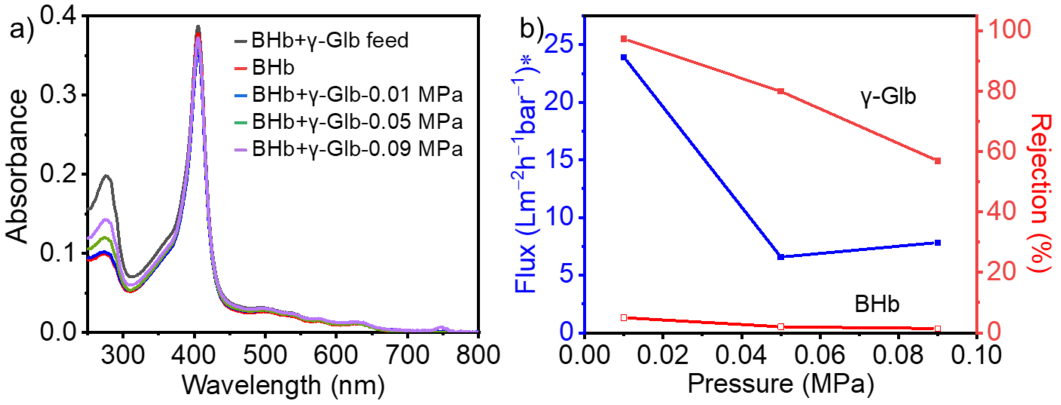

Figure S47. (a) UV-vis spectra of BHb and γ-Glb mixture aqueous solutions before and after separation through membrane M57, and (b) plots of water flux and rejection efficiency versus the change of pressure. "*" means the flux value×100.

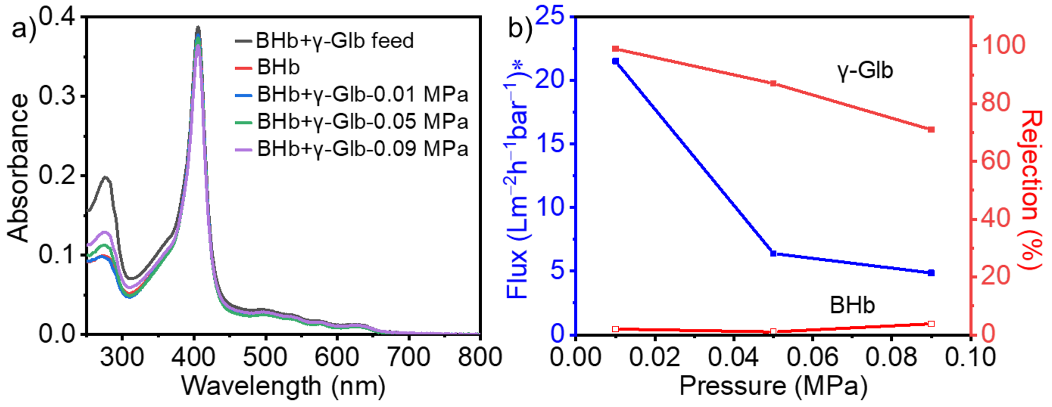

Figure S48. (a) UV-vis spectra of BHb and γ-Glb mixture aqueous solutions before and after separation through membrane M105, and (b) plots of water flux and rejection efficiency versus the change of pressure. "*" means the flux value×100.

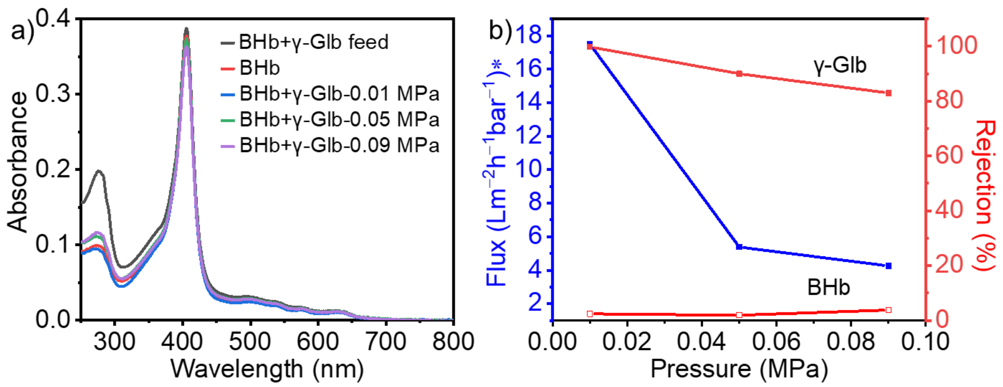

Figure S49. (a) UV-vis spectra of BHb and γ-Glb mixture aqueous solutions before and after separation through membrane M136, and (b) plots of water flux and rejection efficiency versus the change of pressure. "*" means the flux value×100.

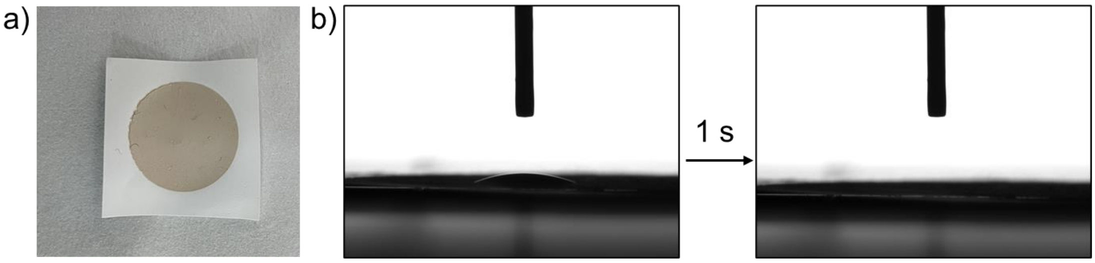

Figure S50. (a) Digital photograph of the 3D SF membrane prepared from DMSO/H_2O (1:1 in volume ratio), and (b) the change of its water contact angle and the change within one second.

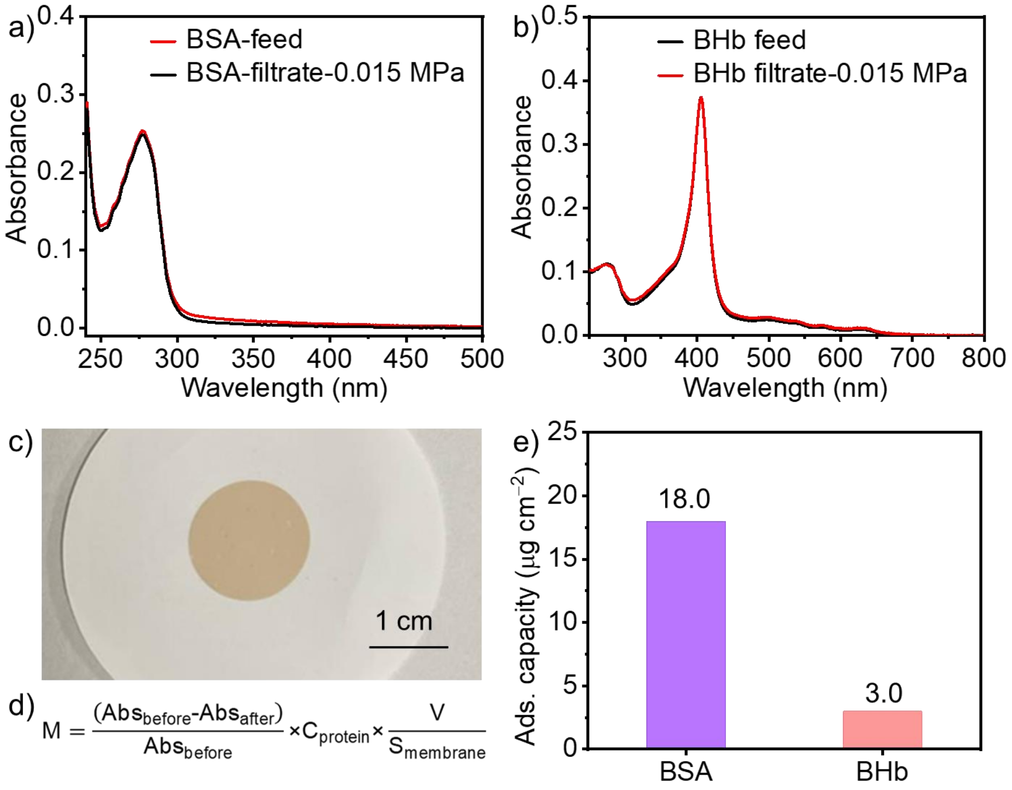

Figure S51. UV-vis spectra of (a) BSA and (b) BHb in aqueous solution before and after filtration through the membrane M136 under a pressure of −0.015 MPa, (c) digital photograph of the filtration membrane, and (d) formula of adsorption capacity, where M is the adsorption capacity (μg cm^{-2}), C is the concentration of proteins (μg mL^{-1}), V denotes the volume of protein solution (5 mL), S means the area of membrane ($S = \pi r^2 = \pi \times 0.75^2$ cm^2), and (e) histogram of adsorption capacity for the two proteins on the used membrane.

S9. Granular 3D SF for enzyme loading and catalysis.

Preparation of HRP@3D SF.

The synthesis of HRP@3D SF was described as follows: 20 mg 3D SF were dispersed in 10 mL water solution with HRP 20 mg, stirring for 12 hours. The obtained precipitate HRP@3D SF was collected by centrifugation. The encapsulation efficiency (~23%) of HRP 3D SF particles was determined by the measurement of the remained HRP in the supernatant, using UV-vis spectroscopy at 403 nm.

Fluorescence labeling of HRP.

20 mg HRP was dispersed into 10 mL sodium carbonate buffer solution (pH=9.0, 0.5 M) and then 1 mg FITC was added. The mixture was stirred in the dark for 12 hours. Finally, the FITC-labelled HRP was obtained by ultrafiltration with centrifugal filter device (molecular weight cut-off MWCO= 8 kDa) for 3 times to remove excess reaction reagents and salts.

Bioactivity of HRP@3D SF.

The bioactivity of HRP@3D SF was measured through the decomposition of hydrogen peroxide, where 3,3',5,5'-Tetramethylbenzidine (TMB) was used as the hydrogen donor.[7−9] The TMB

solution was prepared as follow: 0.15 g TMB was dissolved in 3 mL DMSO, followed by adding 50 mL glycerol solution containing 0.2 g ethylene diamine tetraacetic acid (EDTA), 0.95 g citric acid. Finally, the solution was filled to 500 mL by deionized water.

In a typical test, the HRP@3D SF (1.0 mg) was firstly dispersed in 100 μL sodium dihydrogen phosphate-citric acid buffer (with a pH=7.2). Then, 50 μL H_2O_2 solution (0.01% w/w in deionized water) and 50 μL prepared TMB solution were added, respectively. After 10 min reaction, 10 μL sulfuric acid was introduced to stop the reaction, accompanied by the color changed from blue to luminous yellow. Finally, the produce was immediately monitored at 450 nm using a UV-vis spectrophotometer. The strength of the UV-vis absorption at 450 nm reflected the bioactivity of HRP@3D SF. For evaluating the bioactivities of free HRP, the amount of free enzymes introduced was adjusted to be equal to the amount of enzymes encapsulated into HRP@3D SF (~23 wt% encapsulating efficiency)

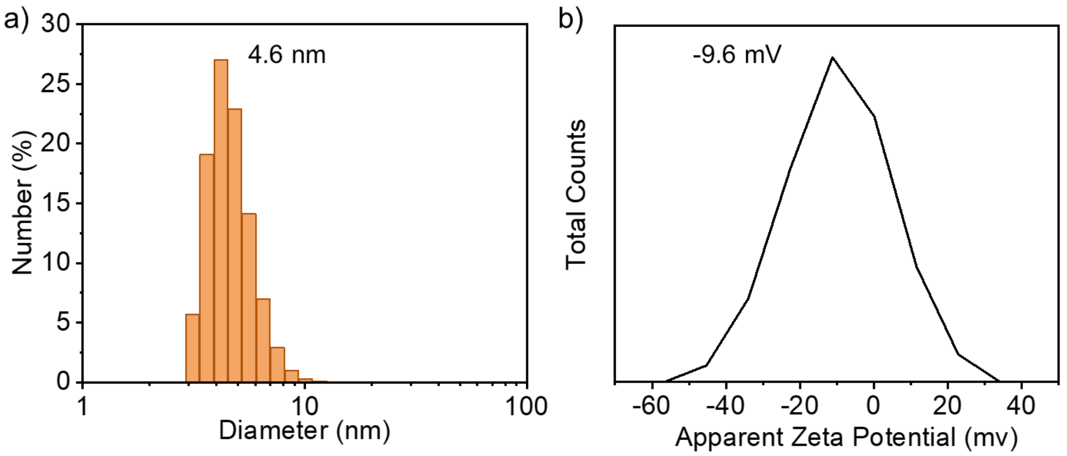

Figure S52. (a) DLS histogram and (b) zeta potential diagram of HRP in water.

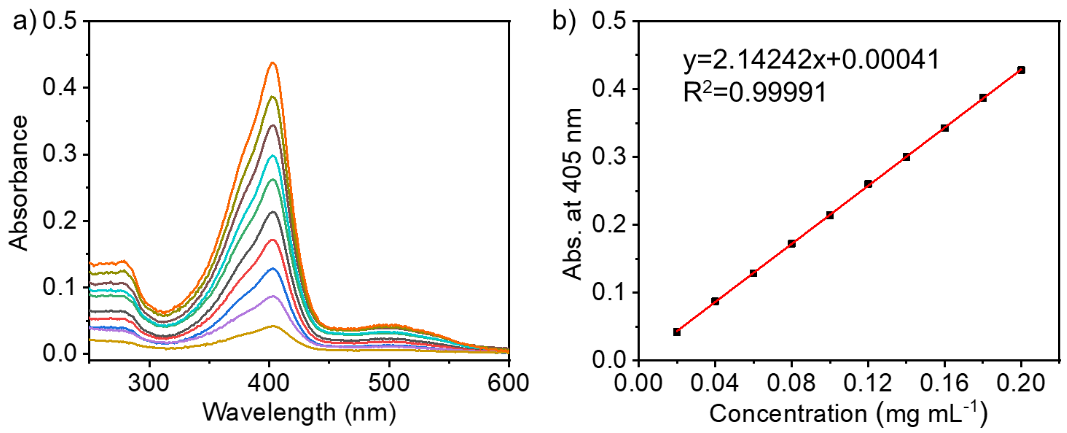

Figure S53. (a) UV-vis spectra of HRP in water at the concentrations of 0.2, 0.18, 0.16, 0.14, 0.12, 0.1, 0.08, 0.06, 0.04, and 0.02 mg mL^{-1} from top to bottom, and (b) the corresponding plot of absorbance at 405 nm upon the concentration.

Table S5. Comparison of the enzymes' loading efficiency of the reported and the present framework materials.

Carrier	Biomolecules	Loading efficiency (w/w %)	Reference
ZIF-8	HRP, MB	14.4% and 18.2%	[10]
CB-UIO-66-Zr	HRP, Laccase, Cellulase	17.1%, 17.6% and 16.9%	[11]
ZIF-8	HRP, GOx, Cyt c	0.59~4.2%	[12]
HBF	HRP, MB, BSA	34.2%, 44.6% and 33.2%	[13]
ZIF-8	HRP, GOx, Cyt c	4.0~8.45%	[14]
MHOFs	HRP, Uox	22.3%, 18.3%	[15]
TpBD	Gox	12.9%	[16]
Tb-mesoMOF	Cyt c	10%	[17]
Granular 3D SF	HRP	23%	This work

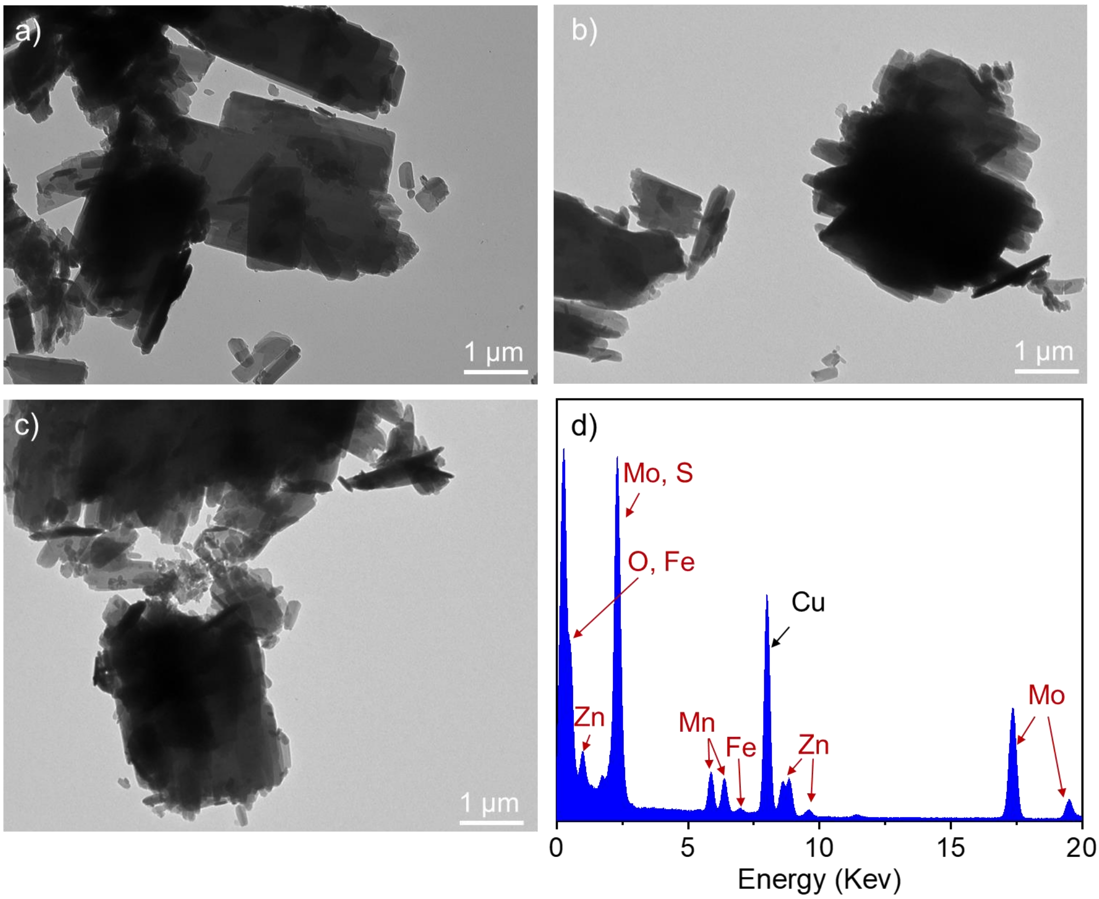

Figure S54. TEM images of 3D SF assembly prepared in water under sonication (a, b) before and (c) after loading HRP, (d) energy dispersive X-ray spectrum (EDX) focusing on the HRP@3D SF assembly.

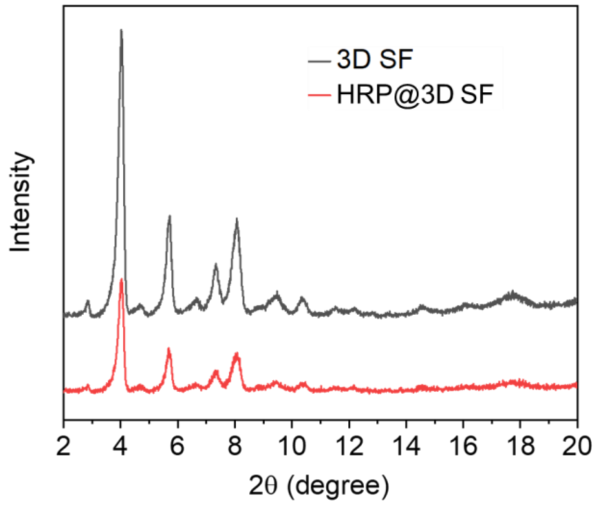

Figure S55. Powder XRD patterns of 3D SF assembly prepared in water under sonication before and after loading HRP.

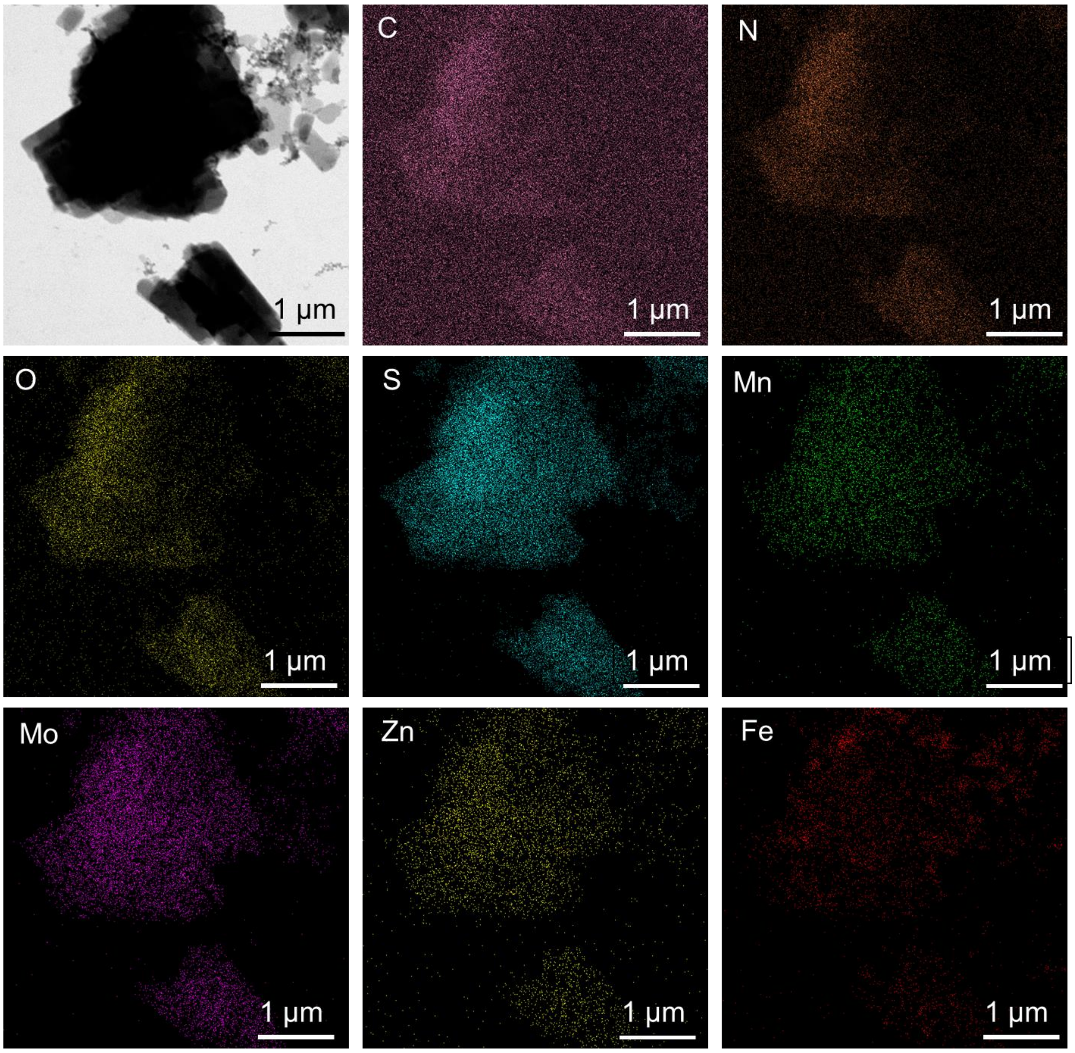

Figure S56. Element mapping images of the obtained granular assemblies indicating the presence of iron, zinc, manganese, and molybdenum elements in HRP@3D SF assembly.

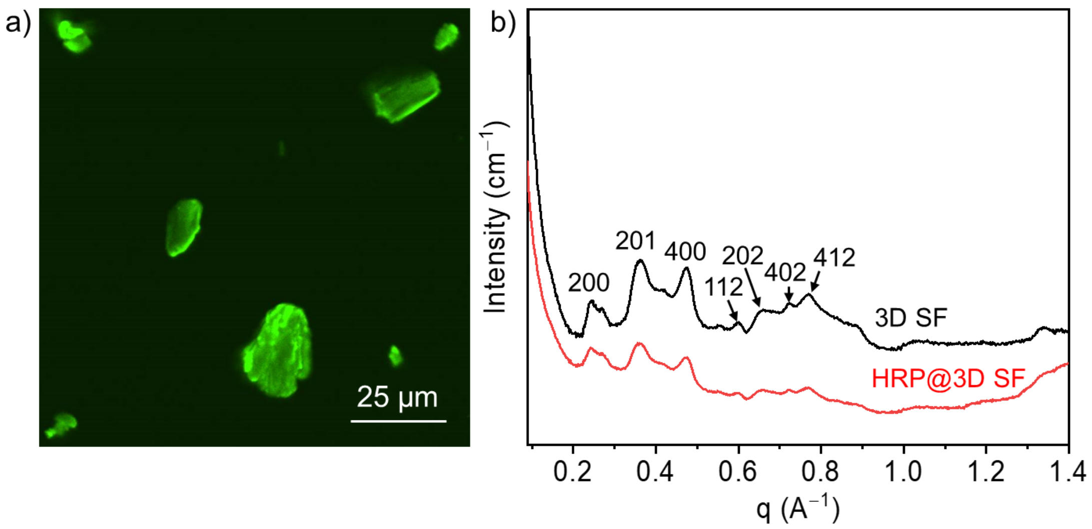

Figure S57. (a) Laser confocal fluorescent microscope image of HRP@3D SF tagged with FITC, and (b) SAXS curves of the 3D SF and HRP@3D SF.

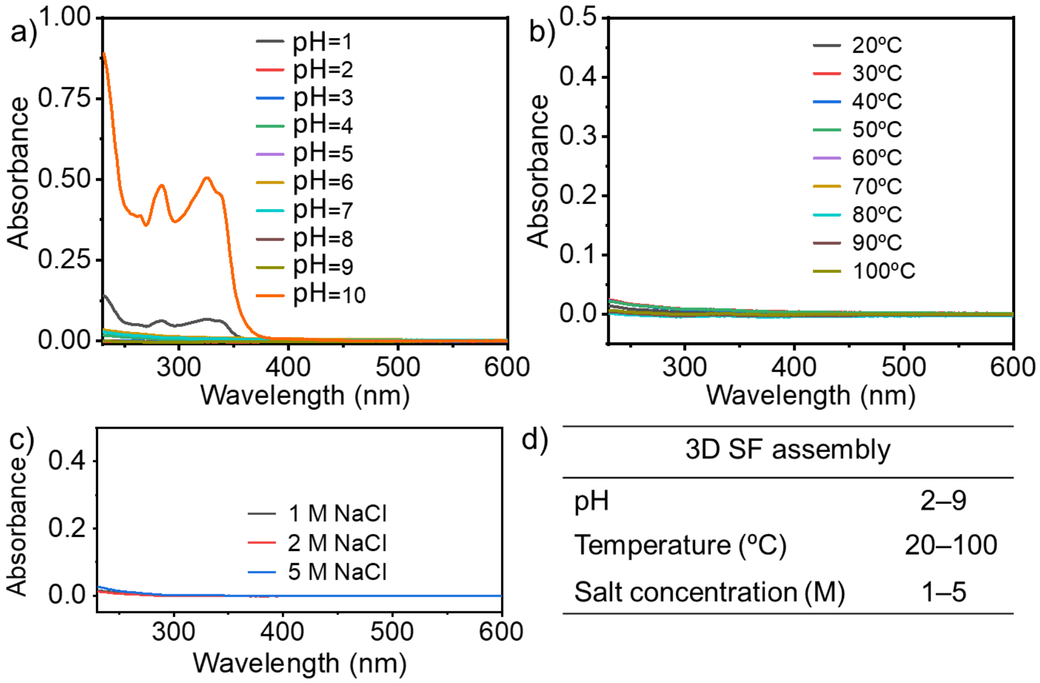

Figure S58. UV-vis spectra of 3D SF membrane versus the change of (a) pH values, (b) temperatures, and (c) salt concentrations in water, and (d) the summary of (a–c).

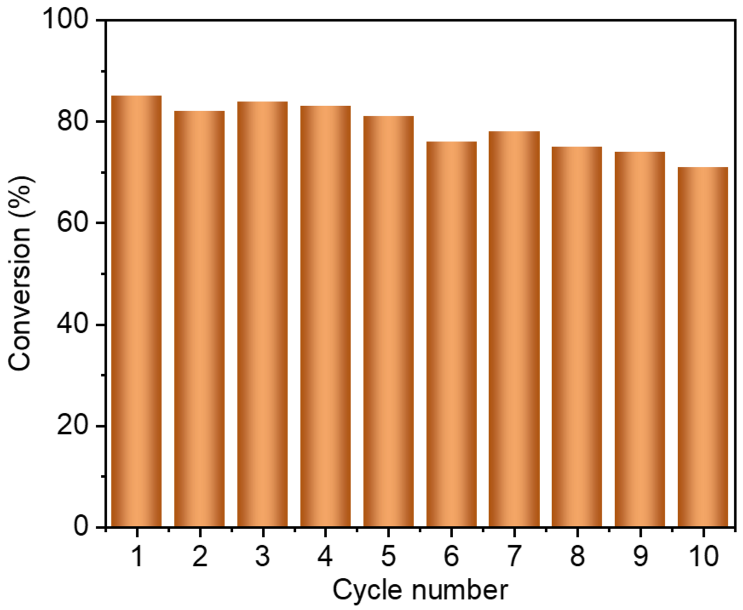

Figure S59. Column graphs of conversion for the catalytic oxidation of TMB by HRP@3D SF carrier under various cycle numbers.

S10. References

[1] H. Z. Yu, X.Y. Qiu, S. P. Nunes, K. v. Peinemann, *Angew. Chem. Int. Ed.* **2014**, *53*, 10072.

[2] H. Xia, Y. Xiahou, P. Zhang, W. C. Ding, D. Y. Wang, *Langmuir* **2016**, *32*, 5870.

[3] Z. Ye, Q. Zhang,S. T. Wang, P. Bharate, S. Varela-Aramburu, M. J. Lu, P. H. Seeberger, J. Yin, *Chem. -Eur. J.* **2016**, *22*, 15216.

[4] R. R. Panicker, A. Sivaramakrishna, *Coord. Chem. Rev.* **2022**, *459*, 214426.

[5] L. Yue, H. Ai, Y. Yang, W. J. Lu , L. X. Wu, *Chem. Commun.* **2013**, *49*, 9770.

[6] C. Yvon, A. J. Surman, M. Hutin, J. Alex, B. O. Smith, D. L. Long, L. Cronin, *Angew. Chem. Int. Ed.* **2014**, *53*, 3336.

[7] S. M. Huang , G. S. Chen, G. F. Ouyang, *Chem. Soc. Rev.* **2022**, *51,* 6824.

[8] Z. J. Mu, Y. H. Zhu, B. X. Li, A. W. Dong, B. Wang, X. Feng, *J. Am. Chem. Soc.* **2022**, *144*, 5145.

[9] J. Y. Song, W. T. He, H. Shen, Z. X. Zhou, M. Q. Li, P. Su, Y. Yang, *Chem. Eng. J.* **2019**, *363*, 174.

[10] G. S. Chen, S. M. Huang, X. X. Kou, S. B. Wei, S. Y. Huang, S. Q. Jiang, J. Shen, F. Zhu, G. F. Ouyang, *Angew. Chem. Int. Ed.* **2019**, *28*, 1463.

[11] J. C. Wu, J. Han, Y. L. Mao, L. Wang, Y. Wang, Y. Y. Li, Y. Wang, *Sep. Purif. Technol.* **2022**, *297*, 121505.

[12] X. L. Wu, H. Yue, Y. Y. Zhang, X. Y. Gao, X. Y. Li, L. C. Wang, Y. F. Cao, M. Hou, H. X. An, L. Zhang, S. Li, J. Y. Ma, H. Lin, Y. A. Fu, H. K. Gu, W. Y. Lou, W. Wei, R. N. Zare, J. Ge, *Nat. Commun.* **2019**, *10*, 5165.

[13] G. S. Chen, S. M. Huang, Y. Shen, X. X. Kou, X. M. Ma, S. Y. Huang, Q. Tong, K. L. Ma, W. Chen, P. Y. Wang, J. Shen, F. Zhu, G. F. Ouyang, *Chem* **2021**, *7*, 2722.

[14] G. S. Chen, S. M. Huang, X. X. Kou, F. Zhu, G. F. Ouyang, *Angew. Chem. Int. Ed.* **2020**, *59*, 13947.
[15] Z. P. Tang, X. Y. Li, L. J. Tong, H. S. Yang, J. Y. Wu, X. L. Zhang, T. Song, S. M. Huang, F. Zhu, G. S. Chen, G. F. Ouyang, *Angew. Chem. Int. Ed.* **2021**, *60*, 23608.
[16] Z. H. Wu, H. T. Shan, Y. S. Jiao, S. Y. Huang, X. D. Wang, K. Liang, J. F. Shi, *Chem. Eur. J.* **2022**, *450*, 138446.
[17] Y. Chen, V. Lykourinou, C. Vetromile, T. Hoang, L. J. Ming, Randy W. Larsen, S. Q. Ma, *J. Am. Chem. Soc.* **2012**, *134*, 13188.
